# Scientific X-ray: Scanning and quantifying the idea evolution of scientific publications

**DOI:** 10.1371/journal.pone.0275192

**Published:** 2022-09-28

**Authors:** Qi Li, Xinbing Wang, Luoyi Fu, Jianghao Wang, Ling Yao, Xiaoying Gan, Chenghu Zhou

**Affiliations:** 1 Department of Electronic Engineering, Shanghai Jiao Tong University, Shanghai, China; 2 Department of Computer Science and Engineering, Shanghai Jiao Tong University, Shanghai, China; 3 State Key Laboratory of Resources and Environmental Information System, Institute of Geographic Sciences and Natural Resources Research, Chinese Academy of Sciences, Beijing, China; University of Bologna, ITALY

## Abstract

The rapid development of modern science nowadays makes it rather challenging to pick out valuable ideas from massive scientific literature. Existing widely-adopted citation-based metrics are not adequate for measuring how well the idea presented by a single publication is developed and whether it is worth following. Here, inspired by traditional X-ray imaging, which returns internal structure imaging of real objects along with corresponding structure analysis, we propose Scientific X-ray, a framework that quantifies the development degree and development potential for any scientific idea through an assembly of ‘X-ray’ scanning, visualization and parsing operated on the citation network associated with a target publication. We pick all 71,431 scientific articles of citation counts over 1,000 as high-impact target publications among totally 204,664,199 publications that cover 16 disciplines spanning from 1800 to 2021. Our proposed Scientific X-ray reproduces how an idea evolves from the very original target publication all the way to the up to date status via an extracted ‘idea tree’ that attempts to preserve the most representative idea flow structure underneath each citation network. Interestingly, we observe that while the citation counts of publications may increase unlimitedly, the maximum valid idea inheritance of those target publications, i.e., the valid depth of the idea tree, cannot exceed a limit of six hops, and the idea evolution structure of any arbitrary publication unexceptionally falls into six fixed patterns. Combined with a development potential index that we further design based on the extracted idea tree, Scientific X-ray can vividly tell how further a given idea presented by a given publication can still go from any well-established starting point. Scientific X-ray successfully identifies 40 out of 49 topics of Nobel prize as high-potential topics by their prize-winning papers in an average of nine years before the prizes are released. Various trials on articles of diverse topics also confirm the power of Scientific X-ray in digging out influential/promising ideas. Scientific X-ray is user-friendly to researchers with any level of expertise, thus providing important basis for grasping research trends, helping scientific policy-making and even promoting social development.

## Introduction

The value of the ideas presented in scientific publications is a critical foundation in the decision-making process for resource allocation [1–3], scientific awards [4] and direction selection [5, 6]. For the governments, high-potential ideas should be funded to allocate trillions of research funding taxed from the public efficiently [7]. For the awarding organizations, ideas with far-reaching impacts need to be picked out to select the prize winners. For the researchers, promising ideas should be followed as a priority to publish more impactful work. However, the volume of scientific literature is proliferating along with the explosion of the scientific enterprise, and it is impossible to read all publications and then select the valuable ideas [8, 9]. This limits the quality of funding, awarding and individual research output, which indirectly slows the advance of scientific enterprise [10, 11]. To fill this gap, the increase of high-quality scientific publication data [2, 7, 12] has significantly attracted interest in utilizing data-driven methods to understand the process of scientific evolution [5, 13].

Citation counts [14, 15] is the most widely used metric for literature evaluation because of its simplicity. But it only captures the low-dimensional structural features of a single publication while ignoring the more complex citation structure associated with it, thus providing a suboptimal result [16–18] for the selection of the optimal idea. The early CD-ROM versions of ISI (Institute of Science Index) databases [19–22] enabled navigation among related scientific literature through shared references, which made it possible to construct citation structure among scientific literature and served as the basis for subsequent academic network analysis. The value of network topology for understanding scientific evolution has been reported in recent work [23–25]. Academic networks are utilized to explore the factors that influence scientific progress in terms of scientific awards [26], technological change [27], and the proliferation of publications [10]. The network science-based approach is also used to understand the relationships between different things, including society and science [28], articles and publishers [3], and knowledge in different disciplines [29, 30]. These methods demonstrate the power of topology among academic entities for predicting scientific trends [6, 9] and even quantifying the reputation of artists [31]. An important role of academic networks is to model knowledge flow. Soler [32] simulates the flow of knowledge through publications on the network and utilizes a function of the number of references and citations to measure the creativity of the target publication. Renoust et al. [33] model the production of knowledge based on the knowledge flow in citation networks. Park and Yoon [34] discover the future direction of technology convergence by predicting potential knowledge flow in citation networks. In the citation network, the main paths of knowledge flow through are also used to trace the development in a specific research field, which is often called the main path analysis [35, 36]. Similar to the main path analysis, we utilize the number of valid layers of knowledge flow in the citation network to quantify the development degree of the target publication and use the fact that knowledge becomes obsolete as it diffuses like the radioactivity of isotope attenuates over time as a basis for assessing the future potential of the target publication.

For the study of scientific literature’s obsolescence, Burton and Kebler first introduced a concept similar to the ‘half-life’ of radioisotopes to describe the aging of scientific publications [37]. They defined the half-life of scientific literature as the time spent in half of the current active publications was published. In 1970, Brooks proposed the classical negative exponential equation for the life cycle of the literature [38]. He found that the decay of paper citation frequency over time approximately obeyed a negative exponential equation. The Price Index is also a well-known indicator for measuring the aging speed of literature [39]. In a specific scientific field, it is defined as the ratio between the citation counts of articles not older than 5 years and the citation counts of all articles. With the continuous research of literature obsolescence, researchers began to correct the calculation of the half-life. Line [40] argues that the concept of half-life proposed by Burton and Kebler is a combination of the literature obsolescence rate and the growth rate and that if the latter is not known, a misleading half-life will be obtained. And the authors obtained the corrected half-life by removing the growth factor from the median citation age. Brown [41] introduces a Poisson distribution to measure obsolescence or reduction of citation counts and tries to further improve the accuracy of the half-life calculation. With the rapid rise of digital information resources, the research on the life cycle of scientific literature has begun to face larger data volumes and richer research objects. Lisée et al. [42] use Thomson’s 1980–2005 CD-ROM data to specifically study the aging characteristics of the papers in conference proceedings. Based on ISI data, Larivière et al. [43] study the aging phenomenon of literature over a very long time from the larger data volume from 1900 to 2004. Wang et al. [44] examine the impact of open access on the aging of journals’ citations. Based on the fact that scientific publications will continue to become obsolete, our framework measures the development potential of the target publication by measuring the aging of its key successors’ knowledge.

There are many excellent tools available for citation analysis through citation network visualization. HisCite [45] and CitNetExplorer [46] can visualize citation relationships between different publications in a field in a hierarchical manner, thus helping researchers to understand the development of the field. CiteSpace [47] can use scientific literature co-citation analysis to reveal the flow of scientific ideas within a field. However, these tools are mostly used for citation analysis of the literature in a specific field, rather than for identifying valuable publications, specifically, for quantifying the development degree and the development potential of the target publications.

**Fig 1:**
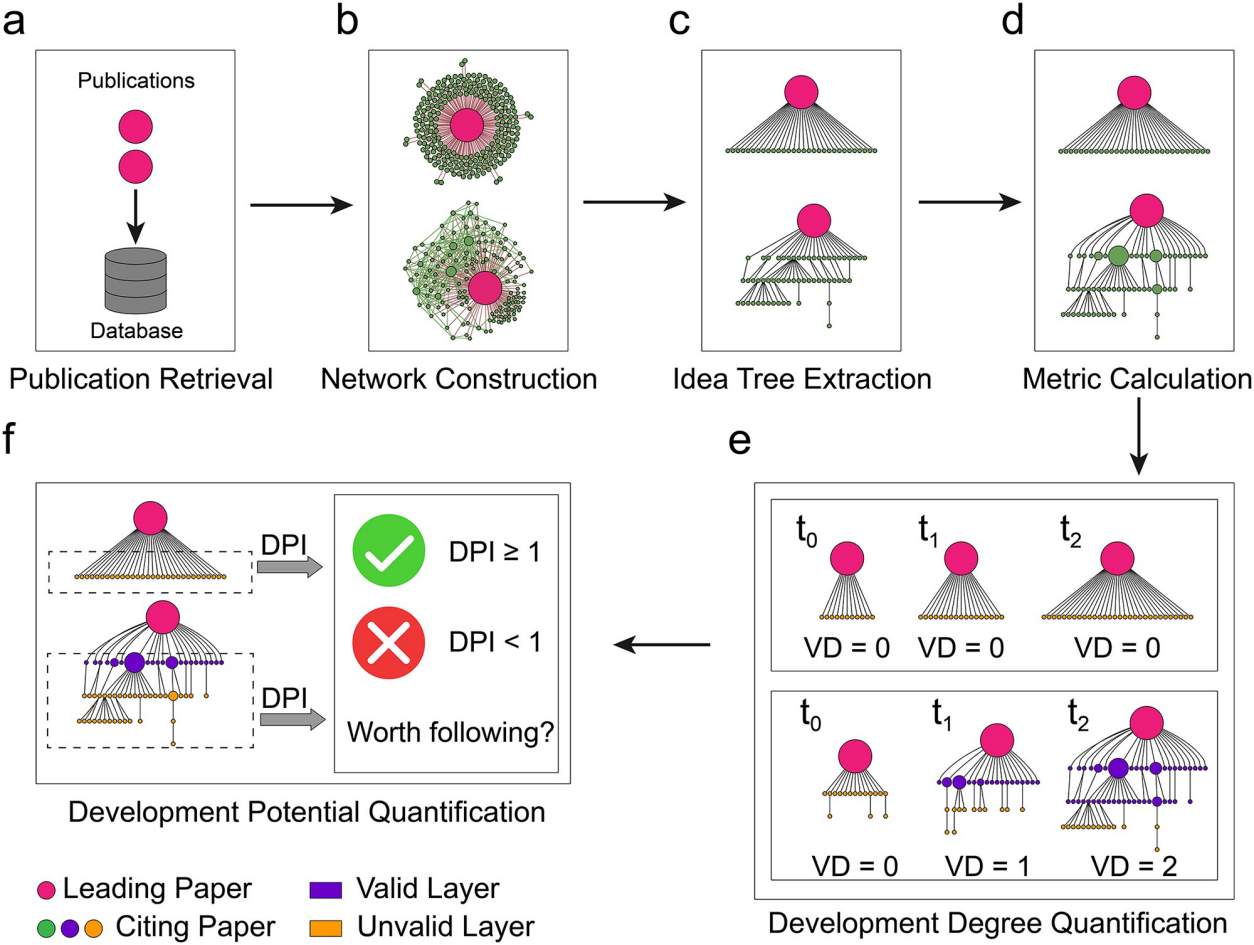
The framework of scientific X-ray. We illustrate the pipeline utilizing two publications with different citation structures. (a) Retrieve the citing papers of the target publication, all links among the target publication and citing articles and all links among citing papers in the database. (b) Construct the citation network of the target publication based on the retrieval results. (c) Extract the idea tree from the citation network to reveal the flow of the target publication’s idea. (d) Utilize Knowledge Entropy (KE) to quantify the knowledge quality of nodes in the idea tree to highlight the powerful inheritor of the target idea. (e) Reproduce the evolution of the idea tree and quantify the degree of development of the target publication’s idea utilizing the Valid Depth (VD). (f) Utilize the Development Potential Index (DPI) to quantify the potential of the target publication and assess whether it is worth continuing to follow.

Here, we propose Scientific X-ray, a framework that scans the citation network associated with the target publication and quantifies the evolution of the target publication’s idea from the visualization result to identify how well the idea is inherited and the potential for future development. Based on the extracted skeleton structure of the idea flow associated with the target publication, Scientific X-ray can quantitatively describe how far the target paper’s idea has evolved and how far it will develop. To complete the X-ray of the literature, we first retrieve the target publications (Fig 1(a)) and then construct the citation networks led by them (Fig 1(b)). Concretely, this type of citation network consists of the single leading paper, the articles citing the leading paper, all links among leading paper and citing articles and all links among citing articles. By observing visualization results of the citation networks leading by high-impact target publications, we find that different types of leading articles’ ideas will make the citing papers associated in different ways. For example, as shown in Fig 1(b), these two networks present two very different structures. Networks led by summative work (e.g., textbooks, surveys and software toolkits) often have the structure shown in the first network. Their networks only form a uniform ring around the leading article (the nodes in the ring do not cite each other and only cite the leading article). The networks led by more innovative research articles usually show a more complex structure as shown in the second network. (See more examples in S3–1 Fig in S1 File). The different network structures reflect the different diffusion patterns of target publications’ ideas. Therefore, inspired by traditional X-ray imaging, we then take the leading article as the root node and decode and visualize the skeleton of idea flow from the citation network (Fig 1(c)), which calls the ‘idea tree’ (i.e., the X-ray of leading publication). Within the idea tree, we define Knowledge Entropy (KE) to quantify the quality of knowledge of articles based on the complexity of the subtree structure associated with them (Fig 1(d)). KE highlights the powerful inheritor of the idea, which allows us to perceive how ideas evolved intuitively. We call the layer in the idea tree that contains nodes whose *KE* ≥ *M* (here M is 10) as the valid layer and define the number of valid layers at time *t* as the Valid Depth (VD) of the target publication at time *t*. Finally, we utilize VD to quantify the development degree of the leading article’s idea (Fig 1(e)) and predict the future VD as Development Potential Index (DPI) of the idea tree to quantify the target idea’s future potential (Fig 1(f)). (See detailed description of Scientific X-ray’s pipeline in the section of Methods and section S2 in S1 File). Our data and code can be found in https://github.com/liqilcn/scientific_x-ray.

## Results

### The existence of a development limit of any scientific publication’s idea

**Fig 2:**
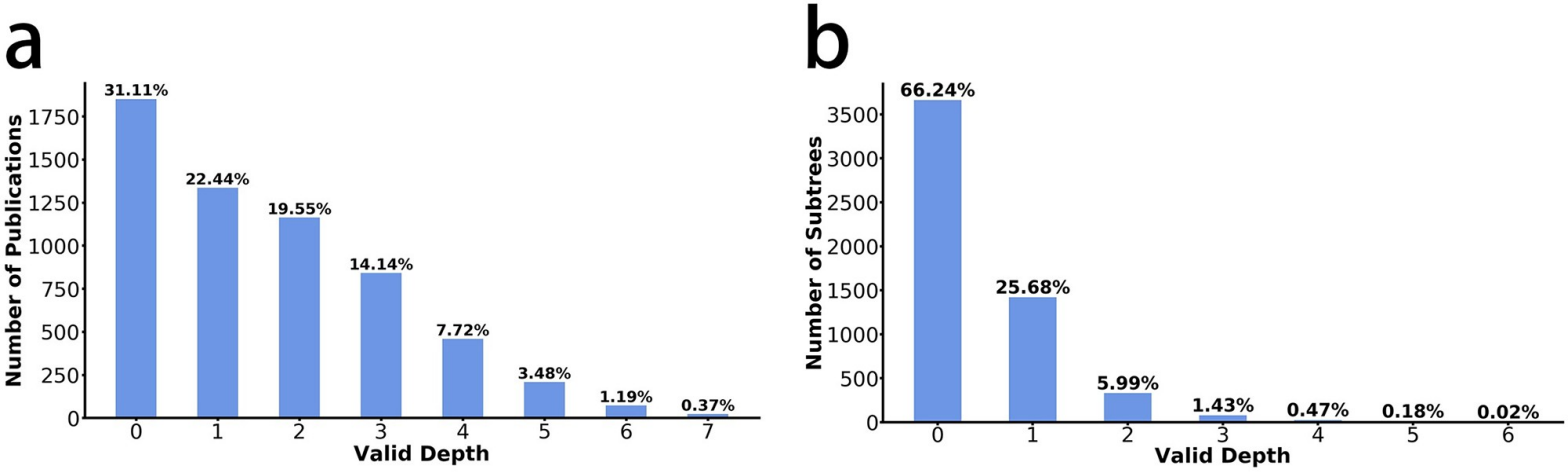
The distribution of high-impact publications’ VD and the distribution of the VD inspired by a single article within idea trees. The VD of 99% of high-impact publications is difficult to exceed six-hop. (b) 99% of the articles in the idea tree have difficulty contributing more than three-hop to the VD.

We utilize Scientific X-ray to analyze the VD of high-impact publications until 2021 (71,431 high-impact publications with *citation*Â *counts* ≥ 1, 000 in the disciplines of History, Computer science, Environmental science, Geology, Psychology, Mathematics, Physics, Materials science, Philosophy, Biology, Medicine, Sociology, Art, Economics, Chemistry, and Political science). We find that even though the citation counts of publications may increase unlimitedly, there is an insurmountable upper bound of the inheritance of the idea: The VD of 99% of scientific publications is difficult to exceed six-hop (Fig 2(a)), which coincides with the ‘Six Degrees of Separation’ theory [48] of psychology and the ‘small-world’ theory [49] of network science. The number of publications with a VD of zero is the largest (31.11%, Fig 2(a)), suggesting that it is difficult for publications to break from zero to one, e.g., new knowledge is difficult to be widely accepted. Different publications may have different VD, which raises a fundamental question: How does the VD evolve? We then explore the evolution patterns of ideas. Among all literature, six representative publications covering the fields of geographic information system, ecology and climate change, computer vision, natural language processing, deep learning and geology have been selected to reveal depth evolution dynamics. (See data details of the corresponding papers in S1–1 and S1–2 Tables in S1 File). We find that idea evolution follows six fixed patterns through our induction and summary, which are widespread in all disciplines.

**Fig 3:**
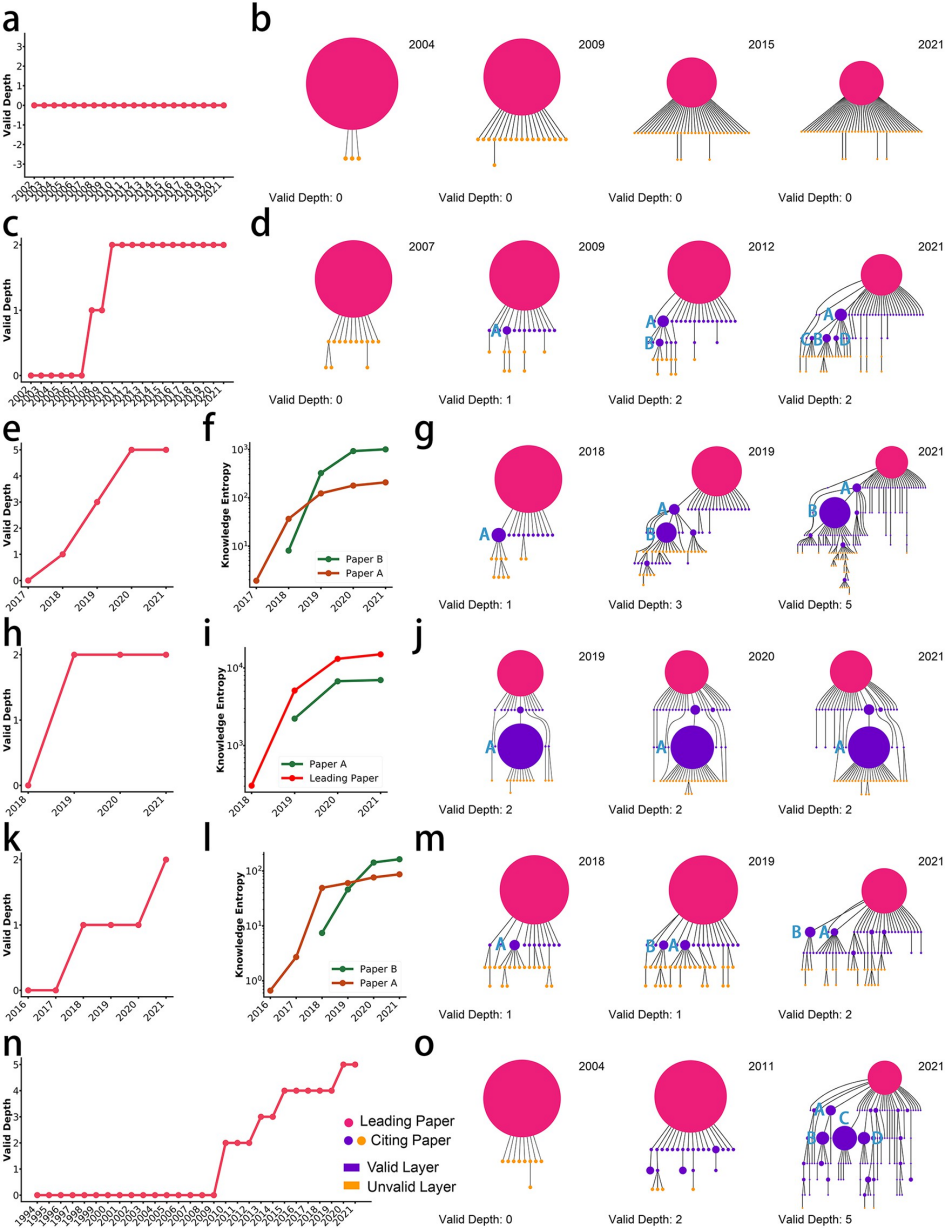
Six fixed evolution patterns of publication’s idea. (a,c,e,h,k,n) The evolution of VD of corresponding publications. (b,d,g,j,m,o) The evolution of idea trees of corresponding publications. All idea trees are pruned to ensure the visibility of the skeleton structure. All idea trees are visualized by the DOT algorithm [50]. Node size is rescaled in every idea tree and positively related to its KE. (f,i,l) The evolution of nodes’ KE in corresponding idea trees. These six patterns exist in a wide range of scientific fields and are not limited to geographic information system, ecology and climate change, computer vision, natural language processing, deep learning and geology.

- **Pattern 1:** *The VD of summative work is hard to exceed one.* The leading work of the idea tree (root node) shown in Fig 3(b) is ‘Geographic Information Systems and Science’, which is a textbook of Geographic Information System (GIS) published in 2001. The leading article’s citation counts grow over time, but its VD does not increase. The leading work of the idea tree tends to summarize existing knowledge instead of proposing new methods or theories, so it is not very inspiring for child nodes and cannot provide new research ideas. We find that almost all idea trees led by summative work such as textbooks, surveys and software toolkits show a similar structure. (See more examples in section S4.1 and S4–1 to S4–4 Figs in S1 File).
- **Pattern 2:** *The increase in VD needs to be driven by non-trivial child nodes.* The leading work of the idea tree shown in Fig 3(d) is ‘Range Shifts and Adaptive Responses to Quaternary Climate Change’. The leading article mainly discusses the impact of climate change on biological evolution. Child article A: ‘Constraint to Adaptive Evolution in Response to Global Warming’ is inspired by the leading article and further discusses the impact of global warming on species evolution. Paper A has attracted much outside attention, making subtree led by it flourish and giving birth to three new high KE articles, including: ‘Predicting the impacts of climate change on the distribution of species: Are bioclimate envelope models useful?’ (Paper B), ‘Evolutionary Responses to Climate Change’ (Paper C) and ‘Adaptation, migration or extirpation: climate change outcomes for tree populations’ (Paper D). All these papers continue to discuss the impact of climate change on biological evolution and attract much attention, which increases the VD to two (Fig 3(c)). Paper A inherits the idea of the leading article and stimulates the generation of new valuable knowledge, thereby promoting the development of the leading article’s idea. (See more examples in section S4.2, S4–5 to S4–8 Figs and S4–1 to S4–4 Tables in S1 File).
- **Pattern 3:** *The continuous increase of the VD needs to be stimulated by the influence relay of multiple high KE nodes.* The leading work of the idea tree shown in Fig 3(g) is ‘Multi-view 3D Object Detection Network for Autonomous Driving’. The leading article creatively proposes the use of multi-class data fusion to improve the accuracy of 3D object detection. Child article A: ‘3D fully convolutional network for vehicle detection in point cloud’ is inspired by the leading article and improves detection accuracy by modifying the model. Its influence started to show in 2018, but the growth of its KE slowed down significantly after 2019. Moreover, at this time, the child node B directly inspired by paper A: ‘Frustum PointNets for 3D Object Detection from RGB-D Data’ further improves the accuracy of 3D object detection by introducing auxiliary information. This allows its KE to surpass that of article A and takes over the task of motivating the VD increase (Fig 3(f)). Inspired by article B, the idea of the leading article can continue to attract outside research interest. This leads to the spawning of several new high KE child nodes under the subtree led by article B and makes the VD continue to increase to five (Fig 3(e)). (See more examples in section S4.3, S4–9 to S4–12 Figs and S4–5 to S4–8 Tables in S1 File).
- **Pattern 4:** *The presence of overpowered child nodes can ruin the increase in the VD.* The leading work of the idea tree shown in Fig 3(j) is ‘Deep contextualized word representations’. The leading article proposes an excellent method to represent words in the text in a high-dimensional vector space. However, the leading article was overshadowed by the emergence of BERT (Article A), which changed the game by moving word representations toward more complex models. The KE of the child article A: ‘BERT: Pre-training of Deep Bidirectional Transformers for Language Understanding’ rapidly increased by over 10^3^ orders of magnitude, thus approaching the order of magnitude of the leading article (Fig 3(i)). This indicates that it has become a new authority, which makes most of the articles citing it do not cite the leading article, and thus the original idea is deprived of new valuable knowledge and eventually stagnates in development(Fig 3(h)). (See more examples in section S4.4, S4–13 to S4–16 Figs and S4–9 to S4–12 Table in S1 File).
- **Pattern 5:** *Stronger branches inhibit the increase in VD of weaker branches.* The leading work of the idea tree shown in Fig 3(m) is ‘DoReFa-Net: Training Low Bitwidth Convolutional Neural Networks with Low Bitwidth Gradients’. The leading work proposes a method to accelerate the training of neural networks in hardware. In the next layer of leading work, two nodes with high KE were born, namely ‘Quantized Neural Networks: Training Neural Networks with Low Precision Weights and Activations’ (Article A) and ‘ShuffleNet: An Extremely Efficient Convolutional Neural Network for Mobile Devices’ (Article B). These two articles formed two factions within the scientific topic. Article A further accelerates the training of neural networks in hardware by compressing the data. Early in the development of the idea tree, the KE by article A was the first to grow (Fig 3(m)). However, article B proposes a very different approach to improving neural networks’ efficiency than article A, which achieves acceleration by designing a more efficient network structure rather than compressing the data. It is the extremely high research and application value of article B that makes its KE surpass that of article B after 2018 (Fig 3(m)), and accordingly, the growth of KE of article A starts to slow down (Fig 3(m)). In this process, article B attracts outside attention, making the branch led by article A neglected, thus causing its development to stagnate. (See more examples in section S4.5, S4–17 to S4–20 Figs and S4–13 to S4–16 Tables in S1 File).
- **Pattern 6:** *VD near the upper bound of development requires a large number of high KE nodes to drive.* The leading work of the idea tree shown in Fig 3(o) is ‘Evolution of the Altaid tectonic collage and Palaeozoic crustal growth in Eurasia’. The leading article proposes a new tectonic model of Eurasia during the Palaeozoic era. Inspired by the leading article, a large number of high KE papers studying the structural evolution of the Central Asian continent have appeared in the idea tree, such as ‘Mesozoic tectonic evolution of the Yanshan fold and thrust belt, with emphasis on Hebei and Liaoning provinces, northern China’ (Paper A), ‘Accretion leading to collision and the Permian Solonker suture, Inner Mongolia, China: Termination of the central Asian orogenic belt’ (Paper B), ‘The Central Asian Orogenic Belt and growth of the continental crust in the Phanerozoic’ (Paper C) and ‘Paleozoic accretionary and collisional tectonics of the eastern Tianshan (China): Implications for the continental growth of central Asia’ (Paper D). The KE of all these articles exceeds 10^2^. Because of this, the VD can reach five (Fig 3(n)), which also reflects the remarkable innovation and inspiration of the leading work’s idea from the side. (See more examples in section S4.6, S4–21 to S4–24 Figs and S4–17 to S4–20 Tables in S1 File).

To prove that the above six patterns can cover most of the evolution patterns of the idea trees, we utilize the rules to automatically divide the evolution patterns of the idea trees. Except for some idea trees where idea inheritance fails to occur between the child nodes, we find that the idea evolution structure of any arbitrary publication unexceptionally falls into the above six fixed patterns. We randomly selected 5,000 publications with *citation*Â *counts* ≥ 1, 000 in the whole academic field and extracted the corresponding idea tree using Scientific X-ray. We automatically classify the idea tree using the rules shown in Table 1.

**Table 1:** The rules and results of the classification of idea trees’ evolution patterns.

Patterns	Rules	Number of patterns
Pattern 1	Keywords (survey, review, summary, introduction, book and software) can be matched in the title of the target publication to indicate that it is a summative article, and the target publication’s VD ≤ 1.	197
Pattern 2	There are high KE child nodes in the subtree led by the high KE node.	1947
Pattern 3	More than three high KE nodes appear on a connected path of the idea tree.	369
Pattern 4	The KE of the child node exceeds the root node, and the VD of the subtree led by it is ≤ 2.	52
Pattern 5	The subtrees led by the two child nodes with KE in the top2 do not contain each other, and among these two nodes, the KE of the first-published node is smaller than the later-published node.	7
Pattern 6	The VD of the target publication reaches five or six, and the number of high KE child nodes exceeds seven.	193

Except for the matched summative articles, we removed all articles with VD = 0 or 1 because the idea trees led by them did not form a complex evolution structure. 2,492 target publications are retained after this step. After automatically classifying these articles using the rules in Table 1, we find that 2,145 articles (86.06%, an article may fall into multiple evolution patterns) fall into the above six patterns. As for the remaining 347 publications, we find that although the VD of the idea trees led by them exceeds one, all the high KE nodes inside the idea tree fail to inspire new high KE nodes. The idea trees led by these articles also fail to form a complex evolution structure. Therefore, except for idea trees that fail to form complex evolution structures, six fixed evolution patterns can cover almost all evolution patterns of idea trees.

The Idea Limit Formula (ILF: ${\Delta D}^{t}\left(v\right)\approx \text{log}\frac{KE^{t}\left(v\right)}{{\left(t-t_{0}\right)}^{1.914}}$, formula (11)) of a single node *v* comes from the observation and induction of the evolution patterns of the idea trees. Similarly, by transforming the value conditions of the ILF, we can uniformly portray the six evolution patterns of the idea tree: (1) For any child node *v*, too small *KE*^*t*^(*v*) cannot advance the idea tree depth to achieve a breakthrough (Δ*D*^*t*^(*v*) is always less than 1), which can explain the Pattern 1 that the summative work cannot breed new high KE nodes and then idea tree is difficult to continue to develop. In order to make VD increase, i.e., to make Δ*D*^*t*^(*v*)≥1, the *KE*^*t*^(*v*) needs to be large enough, which corresponds to Pattern 2. (2) If the nodes with high KE do not promote the increase of the VD within the valid time, its ability to promote VD increase will continue to decay. This can describe Pattern 4 and Pattern 5 where the idea tree has bred high KE nodes, but the VD failed to increase. For Pattern 4, the emergence of overpowered child nodes makes the VD of the idea tree fail to increase for a long time, which causes its Δ*D*^*t*^(*v*) to decay over time, thus making it difficult for the VD of the idea tree to continue to increase. For Pattern 5, although weaker branches can breed high KE nodes in the early stage, they cannot promote the increase of VD for a long time, which will make the promotion effect of high KE nodes continue to weaken. (3) If the high KE nodes appearing in the idea tree are still in the prime time to stimulate the VD, i.e., Δ*D*^*t*^(*v*)≥1, the idea tree is considered to have high development potential, and the VD may keep increasing. Pattern 3 corresponds to the situation that multiple high KE nodes with Δ*D*^*t*^(*v*) ≥ 1 appear continuously on a main line of the idea tree, which enables the VD to continue to increase. Pattern 6 corresponds to the situation where multiple high KE nodes with Δ*D*^*t*^(*v*) ≥ 1 appear on multiple main lines of the idea tree, which makes the VD of the idea tree close to the upper bound of development.

As demonstrated by the patterns above, when high KE articles with different driving effects on VD appear in different positions of idea trees, it will inspire different structures of subtrees. Combining subtrees with different shapes, we can get the idea tree corresponding to the different evolution patterns of ideas. Therefore, we conjecture that the upper development bound of the whole idea tree is attributed to the fact that each child node inside the idea tree has limited capability in driving the VD, and as the depth of the child nodes increases, their ability to drive VD decreases gradually. To verify this conjecture, we randomly select 4,271 idea trees and analyze the driving effect of the high KE child nodes in idea trees. We find that 99% of the articles have difficulty contributing more than three-hop to the VD (Fig 2(b)). We also calculate the mean of VDs driven by high KE child nodes in different Valid Layers (VL). We find that with the increase of depth, the promotion effect of high KE child nodes on VD generally shows a decreasing trend and high KE nodes at layer five or six cannot drive the increase of VD (Table 2). Since it is difficult for any single high KE node inside the idea tree to increase VD to six hops, the driving effect of high KE nodes on VD gradually decays to zero along with the increase of depth, which directly leads to the existence of the upper development bound of the whole idea tree. To this end, the continuing increase in the VD of the idea tree needs to be motivated by the influence relay of multiple high KE nodes, which leads to the emergence of Pattern 3 above. Moreover, when the KE of the article increases to exceed the leading work, due to the destruction effect in Pattern 4, this shifts its effect on the VD from facilitation to inhibition, fundamentally leading to the limit of the depth that a single article can drive.

**Table 2:** Mean of VDs driven by high KE nodes in different valid layers.

Valid Layers	1	2	3	4	5	6
**Mean of VDs**	0.501	0.214	0.128	0.158	0	0

### The verification of scientific X-ray’s VD and DPI by prize data

We utilize top10 advances in science journal’s 2015 breakthrough [51] and Nobel Prize-winning paper dataset [52] to verify the effectiveness of VD and DPI, respectively.

**Fig 4:**
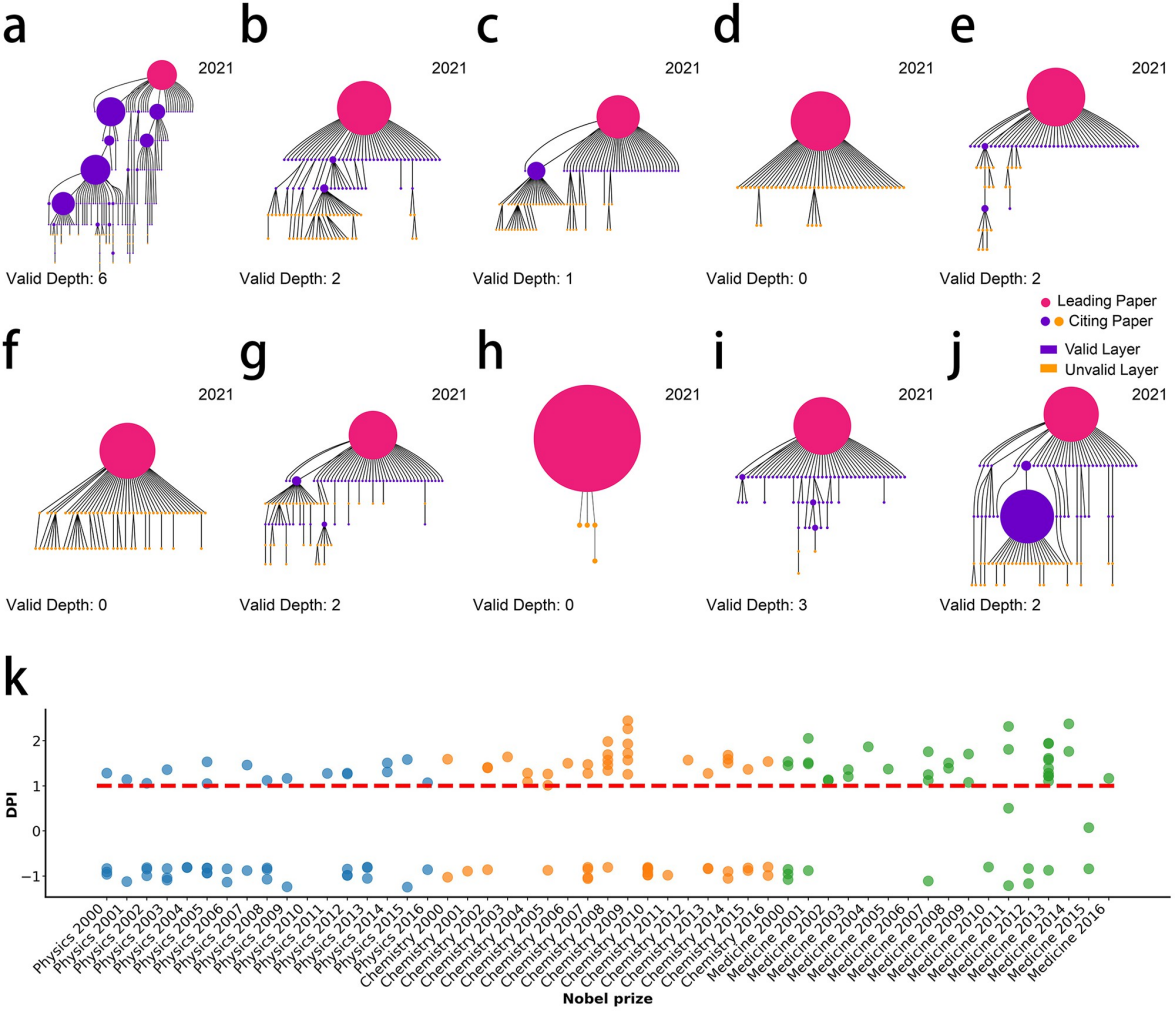
The verification of scientific X-ray’s VD and DPI by prize data. (a) The structure of the idea tree led by ‘A programmable dual-RNA-guided DNA endonuclease in adaptive bacterial immunity’ in 2021 (The Champion of science’s 2015 breakthrough). (b-j) The idea trees of runner-up papers of science’s 2015 breakthrough in 2021. The VD of the idea tree of the champion CRISPR is deeper than other publications, and more high KE nodes appear in the idea tree of CRISPR. (k) Identify the development potential of Nobel topics before they are awarded. Each point represents an award-winning article under the corresponding Nobel Prize topic. The ordinate is the maximum DPI within 1–8 years after the corresponding article was published. Points above the red dotted line are papers with *DPI* ≥ 1 in the time window. Since the average time interval from publication to awarding of prize-winning papers is 17 years, for a Nobel Prize-winning topic, if the maximum DPI of one of the prize-winning articles is over one within 1–8 years after its publication, it is considered that Scientific X-ray has successfully identified corresponding topic’s development potential.

To verify VD’s ability to identify the degree of idea development in even different scientific fields, we collect representative papers of the ten advances in science journal’s 2015 breakthrough and get their X-ray results. (See data details of the corresponding papers in S1–3 and S1–4 Tables in S1 File). The champion of top10 advances is CRISPR (Clustered Regularly Interspaced Short Palindromic Repeats) gene-editing technology. According to the X-ray results until 2021 shown in Fig 4(a)–4(j), compared with other runner-up topics (2–10 of the top10 advances, Fig 4(b) –4(j)), we find that the VD of the seminal paper of CRISPR is the deepest (even reaching six-hop) and the idea tree structure (Fig 4(a)) is the most fruitful. CRISPR was also selected as science’s breakthrough in 2012 and 2013 and won the Nobel Prize in Chemistry in 2020, which further highlights its far-reaching impact beyond other publications. We can intuitively perceive and compare the impact of CRISPR with other disciplines’ topics based on the X-ray results without excessive professional knowledge and other additional analysis.

We then utilize the Nobel Prize-winning paper dataset (including the prizes of Physics, Chemistry, and Physiology or Medicine from 1900 to 2016) to verify the DPI’s ability to identify ideas with development potential. In this dataset, each Nobel Prize topic contains one or more prize-winning papers. We select Nobel Prize topics and their corresponding prize-winning papers from 2000 to 2016 for our experiments and treat each Nobel Prize topic as the topic with development potential before the award. To ensure the generalization capability, the papers in the prize data are not included in the training dataset of ILF fitting. We calculate the DPI of the prize-winning papers before the award to identify the development potential of the related topics. Since the average time interval from publication to awarding of prize-winning papers is 17 years, we set the time interval for analysis to 1 to 8 years after the publication of articles. Scientific X-ray can successfully identify 40/49 Nobel Prize topics (Physics: 13/16, Chemistry: 14/17, and Physiology or Medicine: 13/16) as high potential topics within the time window.

## Discussion

The rapid development of modern science has spawned endless scientific publications, making valuable ideas drown in them and difficult to filter. In this paper, we propose Scientific X-ray, a framework based on the citation structure of a single publication to quantify the degree of development of its idea and its potential for future following. Compared with previous work, Scientific X-ray can visualize the idea flow of a single publication in the form of X-ray and quantify the development degree and future potential of the corresponding idea, enabling the relevant personnel to intuitively identify the idea’s value without the need for excessive expertise. Since Scientific X-ray is based on the citation structures of publications, this enables it to analyze literature in almost all fields.

We utilize Scientific X-ray to list the top10 articles with development potential under the fields of deep learning, geoscience, and Covid-19 (Table 3) (until 2021, lists change over time, see data details of the corresponding papers in S1–5 to S1–10 Tables in S1 File). These top10 papers in these fields are not used to fit ILF to guarantee the generalization capability. The DPI of all these articles is greater than one, which indicates that these papers are still in development. Therefore, after several years, these articles can be used to evaluate the validity of the DPI. In the field of deep learning, the article with the first potential is the seminal paper of Transformer. Transformer originated in the field of natural language processing and is being widely used in the field of computer vision and has gradually developed into a popular general deep learning framework in the field of artificial intelligence. The papers in the second and third positions are both related to few-shot learning. At present, deep learning has two important directions, and the first is to achieve performance improvement by continuously expanding the parameter scale of the model. Scientists have consistently proven that it is feasible to expand the parameter scale to achieve performance improvement. However, this usually comes with a large financial cost and is not the only direction of deep learning. Reducing the dependence of deep learning on data and realizing few-shot learning is another more important research direction. Compared with training large models by hardware stacking to improve performance, few-shot learning can bring higher economic benefit. In the field of geoscience, the first in the list is the research on spatio-temporal data mining of the Covid-19 epidemic published in Science, which indicates a new dynamism in the interdisciplinary research of geoscience and Covid-19. In the emerging field of Covid-19, the researches in the first and second positions are both on drugs used to treat the Covid-19, including Chloroquine and Remdesivir. Moreover, the research in the third position is related to the Covid-19 vaccine (BNT162b2 mRNA Covid-19 Vaccine). This suggests that the urgent issue to be addressed in the research of Covid-19 is how to better treat and prevent Covid-19 through specific drugs and vaccines to overcome the pandemic early. Other studies include the impact of coronavirus on human endothelial cells, the nervous system and the infection manifestations in children and critically ill patients. Scientific X-ray can also be applied to other fields (see other three fields, including computer vision, natural language processing and data mining in section S5 and S5–1 to S5–3 Tables in S1 File), helping people to grasp research trends with little expertise accurately.

**Table 3:** Top ten development potential publications in the field of deep learning, geoscience and Covid-19.

Publication	DPI
***Deep Learning***	
Attention is All you Need	2.935
Prototypical Networks for Few-shot Learning	2.410
Matching networks for one shot learning	2.229
Semi-Supervised Classification with Graph Convolutional Networks	1.843
Understanding deep learning requires rethinking generalization	1.730
A Style-Based Generator Architecture for Generative Adversarial Networks	1.723
Universal Adversarial Perturbations	1.705
Distillation as a Defense to Adversarial Perturbations Against Deep Neural Networks	1.567
DeepFool: A Simple and Accurate Method to Fool Deep Neural Networks	1.427
Overcoming catastrophic forgetting in neural networks	1.376
***Geoscience***	
The effect of human mobility and control measures on the COVID-19 epidemic in China	1.874
Mangroves among the most carbon-rich forests in the tropics	1.746
Global, regional, and national comparative risk assessment of 79 behavioural, environmental and occupational, and metabolic risks or clusters of risks in 188 countries, 1990–2013: a systematic analysis for the Global Burden of Disease Study 2013	1.445
Global land use change, economic globalization, and the looming land scarcity	1.281
‘Structure-from-Motion’ photogrammetry: A low-cost, effective tool for geoscience applications	1.254
Hemispheric and large-scale land-surface air temperature variations: An extensive revision and an update to 2010	1.250
Linear Mixed-Effects Models using‘Eigen’ and S4	1.221
The Transiting Exoplanet Survey Satellite	1.216
Object-based cloud and cloud shadow detection in Landsat imagery	1.170
Bedmap2: improved ice bed, surface and thickness datasets for Antarctica	1.097
***Covid-19***	
Breakthrough: Chloroquine phosphate has shown apparent efficacy in treatment of COVID-19 associated pneumonia in clinical studies	2.741
Compassionate Use of Remdesivir for Patients with Severe Covid-19	2.601
Safety and Efficacy of the BNT162b2 mRNA Covid-19 Vaccine	2.475
Endothelial cell infection and endotheliitis in COVID-19	2.445
Neurologic Manifestations of Hospitalized Patients With Coronavirus Disease 2019 in Wuhan, China	2.137
SARS-CoV-2 Infection in Children	2.110
Characteristics and Outcomes of 21 Critically Ill Patients With COVID-19 in Washington State	2.105
The neuroinvasive potential of SARS-CoV2 may be at least partially responsible for the respiratory failure of COVID-19 patients	2.104
Coronavirus Infections—More Than Just the Common Cold	2.103
The continuing 2019-nCoV epidemic threat of novel coronaviruses to global health—The latest 2019 novel coronavirus outbreak in Wuhan, China	2.046

Scientific X-ray can be further improved in two aspects. First, is the accuracy of the idea tree extraction. A recent study has shown that the Transitive Reduction (TR) of the citation network can effectively remove unimportant citation relationships [53], and incorporating TR in the idea tree extraction may improve the performance of redundant edge removal. Besides, each article in the citation network contains the corresponding semantic information (i.e., title, abstract, etc). in addition to the structure associated with it. We can integrate semantic information into the extraction of the idea tree to improve accuracy. Second, the further improvement of Scientific X-ray also depends on how to measure the knowledge quality of nodes in the idea tree more rationally. The structure related to a single publication is the external expression of the idea under complex interaction mechanisms, and the quality of knowledge contained in an article will not change since its publication. For example, for newly published papers whose citation dynamics have not yet developed, although they may have high development potential, quantitative assessment of their development potential remains a great challenge due to the lack of adequate citation networks associated with the target papers. Therefore, using the content of the paper itself to measure the value of scientific literature will be a challenging and meaningful research direction, which will make the evaluation of literature value independent of citation dynamics and will likely get the value of the literature before the development of citation dynamic.

## Methods

### Data description

Scientific X-ray is built on the database of Acemap [54], which contains 204,664,199 publications (collected and integrated from bibliographic databases, including but not limited to IEEE, ACM, arXiv, Elsevier, and Spring; published from 1800 to 2021; covering 294 fields in 16 disciplines: History, Computer science, Environmental science, Geology, Psychology, Mathematics, Physics, Materials science, Philosophy, Biology, Medicine, Sociology, Art, Economics, Chemistry, and Political science) and their citation relationships. All networks in this paper are constructed from these publications and citation relationships.

### The framework of scientific X-ray

The citation network (Fig 1(b)) formed by the target publication and the citing papers can reflect how the target publication’s idea evolved. Scientific X-ray can decode the idea tree (Fig 1(c)) of the target paper from this type of citation network to reproduce how ideas flow. We define Knowledge Entropy (KE) to quantify the knowledge quality of the nodes in the idea tree to highlight the valid layer of the idea tree (Fig 1(d)). Based on the idea tree, we utilize its Valid Depth (VD) to measure the development degree of the target publication’s idea (Fig 1(e)). To quantify the future value of the target publication’s idea to follow, we define the target publication’s Development Potential Index (DPI) according to its citing papers’ ability to drive VD increase (Fig 1(f)).

### Extracting the idea tree of the target publication

There are too much redundant links in the citation network which has little academic influence on the citing papers. Therefore, repetitive, invalid inheritance relationships need to be removed to clearly and accurately reproduce the flow of the idea, which can be achieved by assessing the similarity between papers. Ideally, we assume that any child article in the network except the leading article is inspired by one of the most essential citation (the more similar the more important) so that we can get an idea tree that reveals the inheritance of ideas. In this way, we can characterize different evolution patterns of ideas through different idea tree structures. There are three steps to extracting the idea tree from the citation network. Initially, the nodes in the network are represented as vectors in a high-dimensional space. Then calculate the reduction index of the node, and measure the importance of the connection according to the difference in the reduction index. Finally, we get the idea tree by cutting the edges between the node pairs which have the largest reduction index difference.

We first measure the distance between the nodes in high dimensional space by graph embedding. As for any target publication, we construct its citation network *G*(*V*, *E*), among which the *V* represents the set of all the nodes and *E* represents the set of all the edges. Especially, *n* = ‖*V*‖ is defined as the number of nodes in the network while *m* = ‖*E*‖ is defined as the number of edges. *A* represents the adjacency matrix of the network, with the form of:

$$\begin{array}{c} A=(\begin{array}{cccc} A_{11} & A_{12} & \ldots & A_{1n}\\ A_{21} & A_{22} & \ldots & A_{2n}\\ \vdots & \vdots & \ddots & \vdots \\ A_{n1} & A_{n2} & \ldots & A_{nn} \end{array}) \end{array}$$ (1)

where *A*_*ij*_ = 1 represents that there exists reference relationship that paper *v*_*i*_ cites paper *v*_*j*_, otherwise, *A*_*ij*_ = 0. We noticed that the target paper cites none of the papers in the network since it’s the earliest one, which blocks our later calculation for eigenvalues and eigenvectors. Considering this, we involve self-citation or self-loop to the target paper, which allows subsequent eigenvalue decomposition. After the process above, we get the adjacency matrix with self-loop *W* where *W*_*ij*_ = *A*_*ij*_ when *v*_*i*_ ≠ *v*_*j*_, *W*_*ij*_ = 0 when *v*_*i*_ = *v*_*j*_ and $\sum_{j,j\neq i}A_{ij}>0$, and *W*_*ij*_ = 1 when *v*_*i*_ = *v*_*j*_ and $\sum_{j,j\neq i}A_{ij}=0$. Continue to process the adjacency matrix with self-loop *W*, we get the out-degree matrix with self-loop *D*:

$$\begin{array}{c} D=(\begin{array}{cccc} d_{1} & 0 & \ldots & 0\\ 0 & d_{2} & \ldots & 0\\ \vdots & \vdots & \ddots & \vdots \\ 0 & 0 & \ldots & d_{n} \end{array}) \end{array}$$ (2)

which is a diagonal matrix with $d_{i}=\sum_{j}W_{ij}$ and *d*_*i*_ ≠ 0. Then we get the normalized Laplace matrix with self-loop:

$$\begin{array}{c} L_{normal}=D^{-\frac{1}{2}}\left(D-W\right)D^{-\frac{1}{2}} \end{array}$$ (3)

Based on this, we perform spectral decomposition of *L*_*normal*_ and get the embedding *eigvector*_*v*_ of any node *v* in the high-dimensional space. Therefore, for any two papers *v*_*i*_ and *v*_*j*_, we can then calculate the distance of the papers in the high-dimensional space.

$$\begin{array}{c} d_{ij}={∥eigvector_{v_{i}}-eigvector_{v_{j}}∥}_{2} \end{array}$$ (4)

Then, to obtain the idea tree by pruning the citation network, we define the reduction index of the node to the entire network to help remove the redundant edges. The reduction index of the node to the entire network characterizes the similarity of the research content of the scientific publication represented by the node to the research content of the whole citation network. Therefore, the greater the difference in the reduction index of two nodes to the entire network, the less similar the research content of these two nodes, and accordingly the less important the edge between them. We first define the reduction index between nodepair. For nodepair (*v*_*i*_, *v*_*j*_), to measure the similarity of research content between *v*_*i*_ and *v*_*j*_, we define the reduction index of nodepair as the sum of the weighted Dijkstra path [55] from *v*_*i*_ to all $v_{j_{k}}$ in *v*_*j*_’s reference list, where the weight is the distance between two adjacent nodes on the path in the high-dimensional space.

$$\begin{array}{c} ReductionIndex_{v_{i},v_{j}}=\sum_{v_{j_{k}}}dijkstra_{v_{i},v_{j_{k}}} \end{array}$$ (5)

Similar to Symeonidis et al. [56], the weighted shortest path is introduced to calculate the similarity between non-neighboring nodes. We are also inspired by the idea that ‘if two nodes are connected to a similar node, then two nodes are similar’ [57]. For article *v*_*j*_, the articles in its reference list can be considered as the source of its ideas. Therefore it can be considered that the closer the article *v*_*i*_ is to the articles in the reference list of *v*_*j*_, the more similar it is to the research content of article *v*_*j*_.

Based on the reduction index of nodepair, for node *v*, its reduction index to the entire network *G* is defined as the sum of its reduction index to all other nodes in the network.

$$\begin{array}{c} ReductionIndex_{v,G}=\sum_{u\in V/v}ReductionIndex_{v,u} \end{array}$$ (6)

The reduction index to the entire network helps us judge the importance of citations. The greater the difference in the reduction index of two nodes to the entire network is, the less important the reference relationship between them is. Therefore, we cut the unimportant citations with the difference in reduction index to the network while maintaining the connectivity in directed graph conditions. Finally, we can get the idea tree from the citation network by keeping the most important citation. (See the detailed description of the algorithm in section S2.1 in S1 File).

### Quantification of articles’ knowledge quality in idea tree

The formation of the structure of the idea tree is driven by nodes with different knowledge quality. Therefore, the effective measurement of knowledge is a fundamental question in revealing the evolution of ideas. For publications, it is almost impossible to go directly, effectively, and uniformly evaluate the quality of knowledge without any experience. Nevertheless, the idea tree preserves the most useful information through a process of de-redundancy, and differences in the complexity of the subtree structure led by a node characterize the difference in the quality of knowledge at that node. We utilize Knowledge Entropy (KE) to quantify the knowledge quality by measuring the complexity of the structure associated with the nodes in the idea tree.

To define the knowledge entropy, we first define the Subtree Entropy of academic paper *a* as follows:

$$\begin{array}{c} H(a)=-\frac{g_{a}}{2m}\text{log}(\frac{V_{a}}{V_{a^{-}}}) \end{array}$$ (7)

The definition of subtree entropy follows the definition of structure entropy in [58], which measures the high-dimensional information embedded in network structures with the help of the partition tree. In the subtree entropy, *g*_*a*_ represents the number of the edges in the original citation network from the nodes in the subtree rooted on *a* in the idea tree to the nodes out of the subtree. *m* represents the number of edges in the idea tree. The larger the *g*_*a*_, the more complex the structure associated with the subtree rooted on *a*. Therefore, the term $\frac{g_{a}}{2m}$ measures the importance of the subtree rooted on *a* to the whole idea tree. *V*_*a*_ represents the number of the nodes in the subtree rooted on *a* while $V_{ab^{-}}$ represents the number of the nodes in the subtree rooted on *a*’s parent node. The term $-\text{log}(\frac{V_{a}}{V_{a^{-}}})$ measures the uncertainty of the subtree rooted on *a* to its parent subtree. Generally speaking, the subtree entropy can measure the effect of the presence or absence of the corresponding subtree on the uncertainty of the whole idea tree. In this case, the greater the influence of a subtree on the idea tree, the greater the subtree entropy.

With the definition of subtree entropy above, the definition of mutual knowledge entropy is also given as follows:

$$\begin{array}{c} I(a,b)=-\frac{g_{ab}}{4m}\text{log}(\frac{V_{a}V_{b}}{V_{{ab}^{-}}^{2}}) \end{array}$$ (8)

where *g*_*ab*_ represents the number of the edges in the original citation network from the nodes in the subtree rooted on *a* and the subtree rooted on *b* in the idea tree to the nodes out of the two subtrees. *m* represents the number of edges in the idea tree. *V*_*a*_ represents the number of the nodes in the subtree rooted on *a*, *V*_*b*_ represents the number of the nodes in the subtree rooted on *b*, while $V_{ab^{-}}$ represents the number of the nodes in the subtree rooted on *a* and *b*’s parent node, which indicates that *a* and *b* should have the same parent node, or the two nodes should locate in similar positions in the idea tree. The mutual knowledge entropy measures the degree of overlap of the knowledge contained in two subtrees. It can be considered that the overlapping knowledge is not created by these subtrees but inherited from the parent node.

Based on the subtree entropy and mutual knowledge entropy above, the definition of knowledge entropy is given as follows: at timestamp *t*, the citation network related to the target publication is *G*^*t*^ = (*V*^*t*^, *E*^*t*^). The idea tree extracted from the network is *IdeaTree*(*G*^*t*^). For any paper *v* belong to *IdeaTree*(*G*^*t*^) except the leading work, we define its knowledge entropy *KE*^*t*^(*v*) as:

$$\begin{array}{c} {KE}^{t}(v)=H^{t}(v)-\sum_{v_{i}\in C^{t}(v)}H^{t}(v_{i})+\sum_{v_{i},v_{j}\in C^{t}(v),i\neq j}I^{t}(v_{i},v_{j}) \end{array}$$ (9)

where *H*^*t*^(*v*) represents the subtree entropy of the subtree led by node *v* at *t*, *C*^*t*^(*v*) represents the children of *v* in *IdeaTree*(*G*^*t*^) at *t*, and *I*^*t*^(*v*_*i*_, *v*_*j*_) represents the mutual subtree entropy of the subtree led by *v*_*i*_ and *v*_*j*_ at *t*. Knowledge entropy is composed of two parts. The first part $H^{t}\left(v\right)-\sum_{v_{i}\in C^{t}\left(v\right)}H^{t}\left(v_{i}\right)$ is the subtree entropy of node *v* minus the subtree entropy of its child nodes, which quantifies the influence of node *v* itself on the formation of the idea tree structure by excluding the influence of child nodes *C*^*t*^(*v*). The first part may be negative, which indicates that the child nodes has more influence on the network structure than the parent node. The second part $\sum_{v_{i},v_{j}\in C^{t}\left(v\right),i\neq j}I^{t}\left(v_{i},v_{j}\right)$ reflects the amount of knowledge inherited by the child nodes *C*^*t*^(*v*) from the parent node *v* utilizing the mutual knowledge entropy from the side. Although the first part of KE may be negative when the knowledge of a parent node is inherited by a large number of child nodes, it causes the second term of the formula to increase, so we still consider it to have high knowledge quality. In the actual calculation, when the second term of the formula is very small, resulting in a negative value of KE, we directly set KE to 0 considering that the amount of knowledge of a scientific article cannot be negative. In this case, the article neither influences the structure sufficiently nor fails to create valuable knowledge to be inherited, so we consider it to have a low knowledge quality. (See the detailed derivation of the formula in section S2.2 in S1 File).

### Quantification of development degree of publication’s idea

The idea of the leading article is developed and refined by attracting the following of high-impact articles. For any child node in the idea tree, each time an idea is inherited along the connection path from the seminal work to this paper, a more complete idea is proposed after referring to the previous ideas. In terms of the whole idea tree, starting from the leading work, the depth of the idea tree represents the number of improvements of the original idea, which characterizes the development degree of the leading article’s idea. Besides, KE helps us to identify it. The appearance of a high KE node at a specific layer of the idea tree symbolizes a qualitative shift of improvement, thus marking a substantial development of the leading article’s idea. Based on this, we call the layer in the idea tree that contains at least one node with knowledge entropy greater than *M* (*M* is 10 in this paper) valid layer. With the evolution of the idea, the deeper layers will gradually become valid. Therefore, based on the idea tree and KE, at timestamp *t*, we define the number of valid layers as the Valid Depth (*VD*^*t*^) of the leading article to characterize the development degree of its idea.

### Quantification of development potential of publication’s idea

We regard the increase in VD due to the combined effect of KE and time decay. The high KE nodes in the idea tree are the key drivers of VD increase. However, for nodes with high KE, their ideas will be continuously propagated and integrated by subsequent work, resulting in their ability to promote VD increase will continue to decay over time. When the effect of KE is stronger than the attenuation of time, the corresponding article will drive the increase in VD. However, the decay effect of time is always present, similar to how the radioactivity of isotope attenuates over time in nature. Since the KE of nodes will not keep increasing, in the long run, the driving effect of high KE nodes will end in the future, and then the development of the idea will come to a standstill. We derive the Idea Limit Formula (ILF) for quantitatively describing the effect of nodes on driving idea tree development based on conjecture, fitting, and verification, which can quantify the development potential of the article within the idea tree.

The form of the idea limit formula is ${\Delta D}^{t}\left(v\right)=\text{log}\frac{KE^{t}\left(v\right)}{{\left(t-t_{0}\right)}^{\gamma }}$, with the help of the least squares method, we use the evolution data of idea tree and knowledge entropy as the sample data to fit the time attenuation coefficient *γ*. For any high knowledge entropy node *v* in any idea tree, node *v* becomes visible at *t*_0_, i.e. $KE^{t_{0}}(v)\geq M$, taking node *v* as a starting point, the valid depth of the subtree led by node *v* is 0 at *t*_0_. We know that the maximum valid depth $MaxVD_{subtree_{v}}$ of the subtree led by node *v* up to the current time *t*_*now*_ is $VD_{subtree_{v}}^{t_{now}}$, at any moment $\bar{t}$ between *t*_0_ and *t*_*now*_, we can get the sample data ${\Delta D}^{\bar{t}}\left(v\right)$ of Δ*D*^*t*^(*v*): ${\Delta D}^{\bar{t}}\left(v\right)=MaxVD_{subtree_{v}}-VD_{subtree_{v}}^{\bar{t}}$. Similarly, we know that the knowledge entropy of node *v* at $\bar{t}$ is $KE^{\bar{t}}\left(v\right)$, therefore, we can get the sample data $\left(KE^{{\bar{t}}_{i}}\left(v_{j}\right),{\bar{t}}_{i}-t_{0},{\Delta D}^{{\bar{t}}_{i}}\left(v_{j}\right)\right)$ from all the idea trees to fit the formula. We transform the form of the formula to log *KE*^*t*^(*v*) − Δ*D*^*t*^(*v*) = *γ* log(*t* − *t*_0_), and the value of *γ* can be obtained according to the least squares method:

$$\begin{array}{c} \hat{\gamma }=\underset{\gamma }{\text{arg}\;\text{min}}\sum_{i,j}{\left(\left(\text{log}KE^{{\bar{t}}_{i}}\left(v_{j}\right)-{\Delta D}^{{\bar{t}}_{i}}\left(v_{j}\right)\right)-\gamma \text{log}\left({\bar{t}}_{i}-t_{0}\right)\right)}^{2} \end{array}$$ (10)

Let the derivative of the objective function to *γ* be 0, we can get $\hat{\gamma }=\frac{\sum_{i,j}\left(\text{log}KE^{{\bar{t}}_{i}}\left(v_{j}\right)-{\Delta D}^{{\bar{t}}_{i}}\left(v_{j}\right)\right)}{\sum_{i,j}\text{log}\left({\bar{t}}_{i}-t_{0}\right)}$, and the fitting result of *γ* is 1.914.

Therefore, assume that the minimum KE of the nodes makes a certain layer of the idea tree valid *KE*_*Threshold*_ = *M*. Node *v* becomes visible at *t*_0_, i.e., $KE^{t_{0}}(v)\geq M$. The depth Δ*D*^*t*^(*v*) that the node *v* can stimulate for the idea tree after time *t* satisfies:

$$\begin{array}{c} {\Delta D}^{t}\left(v\right)\approx \text{log}\frac{KE^{t}\left(v\right)}{{\left(t-t_{0}\right)}^{1.914}} \end{array}$$ (11)

Furthermore, based on the ILF of a single high KE node, we can predict the increase in the VD of the whole idea tree ($\Delta D_{Tree}^{t}$) in the future after the moment *t*. Let *S*^*t*^ be the set of all nodes in the idea tree that meet *KE*^*t*^(*v*) ≥ *M*, the VD of the current idea tree is *VD*^*t*^, for any *v*_*i*_ ∈ *S*^*t*^, its valid layer in the idea tree is $VL_{v_{i}}^{t}$, according to the idea limit formula, the VD that can be stimulated by node *v*_*i*_ for the idea tree after the moment *t* is Δ*D*^*t*^(*v*_*i*_). Therefore, for the whole idea tree, we use $\Delta D_{Tree}^{t}$ to quantify its development potential:

$$\begin{array}{c} \Delta D_{Tree}^{t}=\underset{v_{i}\in S^{t}}{\text{max}}\{\Delta D^{t}(v_{i})-(VD^{t}-VD_{v_{i}}^{t})\} \end{array}$$ (12)

We call $\Delta D_{Tree}^{t}$ the Development Potential Index (DPI) of target publication. Because DPI describes the potential of the idea tree to increase its VD in the future, it is usually compared with one. Publications with DPI greater than or equal to one are considered as articles with development potential, and the greater the DPI, the greater the development potential is.

## Supporting information

S1 File Detailed data descriptions, detailed model descriptions and experiment results. (PDF)
