# Supplementary material for: Scientific X-ray: Scanning and quantifying the idea evolution of scientific publications
Source: PLoS One. 2022 Sep 28;17(9):e0275192. doi: 10.1371/journal.pone.0275192 (PMC9518912; doi:10.1371/journal.pone.0275192)
Supplement: S1 File — (PDF) [file pone.0275192.s001.pdf]

Scientific X-ray: Scanning and quantifying the idea evolution of scientific publications

Qi Li[1◑], Xinbing Wang[2◑*], Luoyi Fu[2◑], Jianghao Wang[3], Ling Yao[3], Xiaoying Gan[1], Chenghu Zhou[3*]

1 Department of Electronic Engineering, Shanghai Jiao Tong University, Shanghai, China
2 Department of Computer Science and Engineering, Shanghai Jiao Tong University, Shanghai, China
3 State Key Laboratory of Resources and Environmental Information System, Institute of Geographic Sciences and Natural Resources Research, Chinese Academy of Sciences, Beijing, China

◑These authors contributed equally to this work.
* xwang8@sjtu.edu.cn (XW); zhouch@lreis.ac.cn (CZ)

Contents

S1 Data details **2**
S1.1 Data in main text 2
S1.2 Data in supplementary materials 9

S2 Model **16**
S2.1 Idea tree extraction algorithm 16
S2.2 The calculation of knowledge entropy 19
S2.3 The fitting of idea limit formula 21

S3 Examples of citation networks pioneered by high-impact publications **22**

S4 Examples of six evolution patterns **23**
S4.1 Pattern 1: The VD of summative work is hard to exceed one 23
S4.2 Pattern 2: The increase in VD needs to be driven by non-trivial child nodes 24
S4.3 Pattern 3: The continuous increase of the VD needs to be stimulated by the influence relay of multiple high KE nodes 28
S4.4 Pattern 4: The presence of overpowered child nodes can ruin the increase in the VD 31
S4.5 Pattern 5: Stronger branches inhibit the increase in VD of weaker branches 34
S4.6 Pattern 6: VD near the upper bound of development requires a large number of high KE nodes to drive 37

S5 Measuring the development potential of publications in specific fields **42**

S1 Data details

Scientific X-ray is built on the database of Acemap, which contains 204,664,199 publications in all disciplines collected and integrated from bibliographic databases, including but not limited to IEEE, ACM, arXiv, Elsevier, and Spring. We select all 71,431 high-impact publications with more than 1,000 citations as pioneering works and construct citation networks for them. All leading articles were published between 1800 and 2021, and their research interests cover 294 fields in 16 disciplines: History, Computer science, Environmental science, Geology, Psychology, Mathematics, Physics, Materials science, Philosophy, Biology, Medicine, Sociology, Art, Economics, Chemistry, and Political science. Data details of the leading articles and citation network overviews appearing in corresponding section of main text and supplementary materials are shown as follows.

S1.1 Data in main text

S1.1.1 The development upper bound of any scientific publication's idea

Leading article	Year	Journal/Conference Series
Geographic Information Systems and Science	2001	Wiley
SphereFace: Deep Hypersphere Embedding for Face Recognition	2017	Computer Vision and Pattern Recognition(CVPR)
Range Shifts and Adaptive Responses to Quaternary Climate Change	2001	Science
DEEP CONTEXTUALIZED WORD REPRESENTATIONS	2018	North American Chapter of the Association for Computational Linguistics(NAACL)
DoReFa-Net: Training Low Bitwidth Convolutional Neural Networks with Low Bitwidth Gradients	2016	arXiv: Neural and Evolutionary Computing
Evolution of the Altaid tectonic collage and Palaeozoic crustal growth in Eurasia	1993	Nature

Table S1-1. Leading article details

Leading article	Nodes	Edges
Geographic Information Systems and Science	1744	2619
SphereFace: Deep Hypersphere Embedding for Face Recognition	1146	4488
Range Shifts and Adaptive Responses to Quaternary Climate Change	1808	8561
DEEP CONTEXTUALIZED WORD REPRESENTATIONS	5215	26245
DoReFa-Net: Training Low Bitwidth Convolutional Neural Networks with Low Bitwidth Gradients	1152	7485
Evolution of the Altaid tectonic collage and Palaeozoic crustal growth in Eurasia	2552	53626

Table S1-2. citation network overviews

S1.1.2 The verification of Scientific X-ray's VD and DPI by prize data

Leading article	Year	Journal/Conference Series
A programmable dual-RNA-guided DNA endonuclease in adaptive bacterial immunity.	2012	Science
The Pluto system: Initial results from its exploration by New Horizons	2015	Science
The ancestry and affiliations of Kennewick Man	2015	Nature
Many psychology papers fail replication test	2015	Science
Homo naledi, a new species of the genus Homo from the Dinaledi Chamber, South Africa	2015	eLife
Broad plumes rooted at the base of the Earth's mantle beneath major hotspots.	2015	Nature
Efficacy and effectiveness of an rVSV-vectored vaccine expressing Ebola surface glycoprotein: interim results from the Guinea ring vaccination cluster-randomised trial	2015	The Lancet
Complete absence of thebaine biosynthesis under home-brew fermentation conditions	2015	bioRxiv
Structural and functional features of central nervous system lymphatic vessels.	2015	Nature
Loophole-free Bell inequality violation using electron spins separated by 1.3 kilometres	2015	Nature

Table S1-3. Leading article details

Leading article	Nodes	Edges
A programmable dual-RNA-guided DNA endonuclease in adaptive bacterial immunity.	7784	134352
The Pluto system: Initial results from its exploration by New Horizons	312	2151
The ancestry and affiliations of Kennewick Man	143	458
Many psychology papers fail replication test	73	83
Homo naledi, a new species of the genus Homo from the Dinaledi Chamber, South Africa	426	1255
Broad plumes rooted at the base of the Earth's mantle beneath major hotspots.	410	1330
Efficacy and effectiveness of an rVSV-vectored vaccine expressing Ebola surface glycoprotein: interim results from the Guinea ring vaccination cluster-randomised trial	577	2240
Complete absence of thebaine biosynthesis under home-brew fermentation conditions	5	5
Structural and functional features of central nervous system lymphatic vessels.	1871	7883
Loophole-free Bell inequality violation using electron spins separated by 1.3 kilometres	1649	7917

Table S1-4. citation network overviews

S1.1.3 Discussion

Deep learning

Leading article	Year	Journal/Conference Series
Attention is All you Need	2017	Neural Information Processing Systems(NeurIPS)
Prototypical Networks for Few-shot Learning	2017	Neural Information Processing Systems(NeurIPS)
Matching networks for one shot learning	2016	Neural Information Processing Systems(NeurIPS)
Semi-Supervised Classification with Graph Convolutional Networks	2017	International Conference on Learning Representations(ICLR)
Understanding deep learning requires rethinking generalization	2017	International Conference on Learning Representations(ICLR)
A Style-Based Generator Architecture for Generative Adversarial Networks.	2018	Computer Vision and Pattern Recognition(CVPR)
Universal Adversarial Perturbations	2017	Computer Vision and Pattern Recognition(CVPR)
Distillation as a Defense to Adversarial Perturbations Against Deep Neural Networks	2016	IEEE Symposium on Security and Privacy(S&P)
DeepFool: A Simple and Accurate Method to Fool Deep Neural Networks	2016	Computer Vision and Pattern Recognition(CVPR)
Overcoming catastrophic forgetting in neural networks	2017	Proceedings of the National Academy of Sciences of the United States of America

Table S1-5. Leading article details

Leading article	Nodes	Edges
Attention is All you Need	15522	83563
Prototypical Networks for Few-shot Learning	2310	14410
Matching networks for one shot learning	1795	9672
Semi-Supervised Classification with Graph Convolutional Networks	3514	14254
Understanding deep learning requires rethinking generalization	1569	6813
A Style-Based Generator Architecture for Generative Adversarial Networks.	1837	6715
Universal Adversarial Perturbations	1236	7888
Distillation as a Defense to Adversarial Perturbations Against Deep Neural Networks	1531	11567
DeepFool: A Simple and Accurate Method to Fool Deep Neural Networks	2328	18905
Overcoming catastrophic forgetting in neural networks	1648	10101

Table S1-6. citation network overviews

Geoscience

Leading article	Year	Journal/Conference Series
The effect of human mobility and control measures on the COVID-19 epidemic in China	2020	Science
Mangroves among the most carbon-rich forests in the tropics	2011	Nature Geoscience
Global, regional, and national comparative risk assessment of 79 behavioural, environmental and occupational, and metabolic risks or clusters of risks in 188 countries, 1990–2013: a systematic analysis for the Global Burden of Disease Study 2013	2015	The Lancet
Global land use change, economic globalization, and the looming land scarcity	2011	Proceedings of the National Academy of Sciences of the United States of America
Hemispheric and large-scale land-surface air temperature variations: An extensive revision and an update to 2010	2012	Journal of Geophysical Research
Linear Mixed-Effects Models using 'Eigen' and S4	2015	Journal of Statistical Software
The Transiting Exoplanet Survey Satellite	2014	arXiv: Earth and Planetary Astrophysics
'Structure-from-Motion' photogrammetry: A low-cost, effective tool for geoscience applications	2012	Geomorphology
Object-based cloud and cloud shadow detection in Landsat imagery	2012	Remote Sensing of Environment
Bedmap2: improved ice bed, surface and thickness datasets for Antarctica	2012	The Cryosphere

Table S1-7. Leading article details

Leading article	Nodes	Edges
The effect of human mobility and control measures on the COVID-19 epidemic in China	1024	2782
Mangroves among the most carbon-rich forests in the tropics	1283	9708
Global, regional, and national comparative risk assessment of 79 behavioural, environmental and occupational, and metabolic risks or clusters of risks in 188 countries, 1990–2013: a systematic analysis for the Global Burden of Disease Study 2013	1430	4036
Global land use change, economic globalization, and the looming land scarcity	1647	5552
Hemispheric and large-scale land-surface air temperature variations: An extensive revision and an update to 2010	1537	5990
Linear Mixed-Effects Models using 'Eigen' and S4	5945	9040
The Transiting Exoplanet Survey Satellite	1464	9277
'Structure-from-Motion' photogrammetry: A low-cost, effective tool for geoscience applications	1747	8570
Object-based cloud and cloud shadow detection in Landsat imagery	1072	5312
Bedmap2: improved ice bed, surface and thickness datasets for Antarctica	1207	9363

Table S1-8. citation network overviews

Covid-19

Leading article	Year	Journal/Conference Series
Breakthrough: Chloroquine phosphate has shown apparent efficacy in treatment of COVID-19 associated pneumonia in clinical studies	2020	BioScience Trends
Compassionate Use of Remdesivir for Patients with Severe Covid-19	2020	The New England Journal of Medicine
Safety and Efficacy of the BNT162b2 mRNA Covid-19 Vaccine.	2020	The New England Journal of Medicine
Endothelial cell infection and endotheliitis in COVID-19	2020	The Lancet
Neurologic Manifestations of Hospitalized Patients With Coronavirus Disease 2019 in Wuhan, China	2020	JAMA Neurology
SARS-CoV-2 Infection in Children	2020	The New England Journal of Medicine
Characteristics and Outcomes of 21 Critically Ill Patients With COVID-19 in Washington State	2020	JAMA
The neuroinvasive potential of SARS-CoV2 may be at least partially responsible for the respiratory failure of COVID-19 patients	2020	Journal of Medical Virology
Coronavirus Infections—More Than Just the Common Cold	2020	JAMA
The continuing 2019-nCoV epidemic threat of novel coronaviruses to global health - The latest 2019 novel coronavirus outbreak in Wuhan, China.	2020	International Journal of Infectious Diseases

Table S1-9. Leading article details

Leading article	Nodes	Edges
Breakthrough: Chloroquine phosphate has shown apparent efficacy in treatment of COVID-19 associated pneumonia in clinical studies	1542	6606
Compassionate Use of Remdesivir for Patients with Severe Covid-19	1616	4185
Safety and Efficacy of the BNT162b2 mRNA Covid-19 Vaccine.	3374	16024
Endothelial cell infection and endotheliitis in COVID-19	2801	12545
Neurologic Manifestations of Hospitalized Patients With Coronavirus Disease 2019 in Wuhan, China	3173	18791
SARS-CoV-2 Infection in Children	1340	5220
Characteristics and Outcomes of 21 Critically Ill Patients With COVID-19 in Washington State	1285	4408
The neuroinvasive potential of SARS-CoV2 may be at least partially responsible for the respiratory failure of COVID-19 patients	1211	5898
Coronavirus Infections—More Than Just the Common Cold	1056	2234
The continuing 2019-nCoV epidemic threat of novel coronaviruses to global health - The latest 2019 novel coronavirus outbreak in Wuhan, China.	1664	3776

Table S1-10. citation network overviews

S1.2 Data in supplementary materials

S1.2.1 Examples of citation networks pioneered by high-impact publications

Leading article	Year	Journal/Conference Series
Applied Predictive Modeling	2018	World Book Inc
A survey of deep neural network architectures and their applications	2017	Neurocomputing
PyTorch: An Imperative Style, High-Performance Deep Learning Library	2019	Neural Information Processing Systems(NeurIPS)

Table S1-11. Leading article details

Leading article	Nodes	Edges
Applied Predictive Modeling	1622	2342
A survey of deep neural network architectures and their applications	1181	1486
PyTorch: An Imperative Style, High-Performance Deep Learning Library	5002	6591
Improved Training of Wasserstein GANs	2053	7522
FlowNet 2.0: Evolution of Optical Flow Estimation with Deep Networks	1471	5264
Context Encoders: Feature Learning by Inpainting	1019	5210

Table S1-12. citation network overviews

S1.2.2 Examples of six evolution patterns

Leading article	Year	Journal/Conference Series
Principal component analysis: a review and recent developments.	2016	Philosophical Transactions of the Royal Society A
Multilevel Analysis : Techniques and Applications, Third Edition	2017	Routledge
An Introduction to Medical Statistics	1987	Oxford University Press
TensorFlow: a system for large-scale machine learning	2016	Operating Systems Design and Implementation(OSDI)
Recent Arctic amplification and extreme mid-latitude weather	2014	Nature Geoscience
Processing seismic ambient noise data to obtain reliable broad-band surface wave dispersion measurements	2007	Geophysical Journal International
Overcoming catastrophic forgetting in neural networks	2017	Proceedings of the National Academy of Sciences of the United States of America
Globally and locally consistent image completion	2017	ACM Transactions on Graphics
Improved techniques for training GANs	2016	Neural Information Processing Systems(NeurIPS)
Matching networks for one shot learning	2016	Neural Information Processing Systems(NeurIPS)
A Meta-Analysis of Global Urban Land Expansion	2011	PLOS ONE
Cleavage of GSDMD by inflammatory caspases determines pyroptotic cell death	2015	Nature

Table S1-13. Leading article details

Leading article	Year	Journal/Conference Series
The Kinetics Human Action Video Dataset	2017	arXiv: Computer Vision and Pattern Recognition
Estimating Corn Leaf Chlorophyll Concentration from Leaf and Canopy Reflectance	2000	Remote Sensing of Environment
Thermal remote sensing of urban climates	2003	Remote Sensing of Environment
Weyl Semimetal Phase in Noncentrosymmetric Transition-Metal Monophosphides	2015	Physical Review X
Non-ideal interactions in calcic amphiboles and their bearing on amphibole-plagioclase thermometry	1994	Contributions to Mineralogy and Petrology
A dynamic global vegetation model for studies of the coupled atmosphere-biosphere system	2005	Global Biogeochemical Cycles
Robust Responses of the Hydrological Cycle to Global Warming	2006	Journal of Climate
Prototypical Networks for Few-shot Learning	2017	Neural Information Processing Systems(NeurIPS)
Unsupervised Representation Learning with Deep Convolutional Generative Adversarial Networks	2016	International Conference on Learning Representations(ICLR)
Feature Pyramid Networks for Object Detection	2017	Computer Vision and Pattern Recognition(CVPR)
Inception-v4, Inception-ResNet and the Impact of Residual Connections on Learning	2016	National Conference on Artificial Intelligence(AAAI)
High Serum IgG4 Concentrations in Patients with Sclerosing Pancreatitis	2001	The New England Journal of Medicine

Table S1-14. Leading article details

Leading article	Nodes	Edges
Principal component analysis: a review and recent developments.	1430	1545
Multilevel Analysis : Techniques and Applications, Third Edition	2322	3268
An Introduction to Medical Statistics	2180	2730
TensorFlow: a system for large-scale machine learning	5941	9676
Recent Arctic amplification and extreme mid-latitude weather	1037	6187
Processing seismic ambient noise data to obtain reliable broad-band surface wave dispersion measurements	1308	10856
Overcoming catastrophic forgetting in neural networks	1648	10101
Globally and locally consistent image completion	1042	4747
Improved techniques for training GANs	2394	17336
Matching networks for one shot learning	1795	9672
A Meta-Analysis of Global Urban Land Expansion	1119	3031
Cleavage of GSDMD by inflammatory caspases determines pyroptotic cell death	1584	14187
The Kinetics Human Action Video Dataset	1202	6373
Estimating Corn Leaf Chlorophyll Concentration from Leaf and Canopy Reflectance	1282	7440
Thermal remote sensing of urban climates	1540	12323
Weyl Semimetal Phase in Noncentrosymmetric Transition-Metal Monophosphides	1162	14237
Non-ideal interactions in calcic amphiboles and their bearing on amphibole-plagioclase thermometry	1661	7493
A dynamic global vegetation model for studies of the coupled atmosphere-biosphere system	1522	10236
Robust Responses of the Hydrological Cycle to Global Warming	3151	24511
Prototypical Networks for Few-shot Learning	2310	14410
Unsupervised Representation Learning with Deep Convolutional Generative Adversarial Networks	6492	50667
Feature Pyramid Networks for Object Detection	5896	36669
Inception-v4, Inception-ResNet and the Impact of Residual Connections on Learning	3551	11192
High Serum IgG4 Concentrations in Patients with Sclerosing Pancreatitis	2056	33255

Table S1-15. citation network overviews

S1.2.3 Measuring the development potential of publications in specific fields

Computer vision

Leading article	Year	Journal/Conference Series
Rethinking Atrous Convolution for Semantic Image Segmentation	2017	arXiv: Computer Vision and Pattern Recognition
PointNet++: Deep Hierarchical Feature Learning on Point Sets in a Metric Space	2017	Neural Information Processing Systems(NeurIPS)
The SYNTHIA Dataset: A Large Collection of Synthetic Images for Semantic Segmentation of Urban Scenes	2016	Computer Vision and Pattern Recognition(CVPR)
ArcFace: Additive Angular Margin Loss for Deep Face Recognition	2018	Computer Vision and Pattern Recognition(CVPR)
YOLO9000: Better, Faster, Stronger	2017	Computer Vision and Pattern Recognition(CVPR)
You Only Look Once: Unified, Real-Time Object Detection	2016	Computer Vision and Pattern Recognition(CVPR)
Deformable Convolutional Networks	2017	International Conference on Computer Vision(ICCV)
Image Super-Resolution Using Deep Convolutional Networks	2016	IEEE Transactions on Pattern Analysis and Machine Intelligence
Dynamic Graph CNN for Learning on Point Clouds	2019	ACM Transactions on Graphics
Image-to-Image Translation with Conditional Adversarial Networks	2017	Computer Vision and Pattern Recognition(CVPR)

Table S1-16. Leading article details

Leading article	Nodes	Edges
Rethinking Atrous Convolution for Semantic Image Segmentation	2419	9098
PointNet++: Deep Hierarchical Feature Learning on Point Sets in a Metric Space	2563	20653
The SYNTHIA Dataset: A Large Collection of Synthetic Images for Semantic Segmentation of Urban Scenes	1131	6776
ArcFace: Additive Angular Margin Loss for Deep Face Recognition	1223	3491
YOLO9000: Better, Faster, Stronger	5027	17497
You Only Look Once: Unified, Real-Time Object Detection	9007	40418
Deformable Convolutional Networks	1582	6907
Image Super-Resolution Using Deep Convolutional Networks	3483	20884
Dynamic Graph CNN for Learning on Point Clouds	1136	4954
Image-to-Image Translation with Conditional Adversarial Networks	7456	38130

Table S1-17. citation network overviews

Natural language processing

Leading article	Year	Journal/Conference Series
A BROAD-COVERAGE CHALLENGE CORPUS FOR SENTENCE UNDERSTANDING THROUGH INFERENCE	2018	North American Chapter of the Association for Computational Linguistics(NAACL)
Get To The Point: Summarization with Pointer-Generator Networks	2017	Meeting of the Association for Computational Linguistics(ACL)
Enriching Word Vectors with Subword Information	2017	Transactions of the Association for Computational Linguistics
SQuAD: 100,000+ Questions for Machine Comprehension of Text	2016	Empirical Methods in Natural Language Processing(EMNLP)
Listen, attend and spell: A neural network for large vocabulary conversational speech recognition	2016	International Conference on Acoustics, Speech, and Signal Processing(ICASSP)
Google's Neural Machine Translation System: Bridging the Gap between Human and Machine Translation	2016	arXiv: Computation and Language
Neural Machine Translation of Rare Words with Subword Units	2016	Meeting of the Association for Computational Linguistics(ACL)
Improving Neural Machine Translation Models with Monolingual Data	2016	Meeting of the Association for Computational Linguistics(ACL)
BERT: Pre-training of Deep Bidirectional Transformers for Language Understanding	2018	arXiv: Computation and Language
XLNet: Generalized Autoregressive Pretraining for Language Understanding	2019	arXiv: Computation and Language

Table S1-18. Leading article details

Leading article	Nodes	Edges
A BROAD-COVERAGE CHALLENGE CORPUS FOR SENTENCE UNDERSTANDING THROUGH INFERENCE	1220	7839
Get To The Point: Summarization with Pointer-Generator Networks	1863	9758
Enriching Word Vectors with Subword Information	4451	12017
SQuAD: 100,000+ Questions for Machine Comprehension of Text	1160	8290
Listen, attend and spell: A neural network for large vocabulary conversational speech recognition	1231	4746
Google's Neural Machine Translation System: Bridging the Gap between Human and Machine Translation	3275	11551
Neural Machine Translation of Rare Words with Subword Units	3749	25844
Improving Neural Machine Translation Models with Monolingual Data	1306	7073
BERT: Pre-training of Deep Bidirectional Transformers for Language Understanding	6898	20124
XLNet: Generalized Autoregressive Pretraining for Language Understanding	1519	4564

Table S1-19. citation network overviews

Data mining

Leading article	Year	Journal/Conference Series
Inductive Representation Learning on Large Graphs	2017	Neural Information Processing Systems(NeurIPS)
Wide & Deep Learning for Recommender Systems	2016	Conference on Recommender Systems(RecSys)
Membership Inference Attacks Against Machine Learning Models	2017	IEEE Symposium on Security and Privacy(S&P)
Deep Neural Networks for YouTube Recommendations	2016	Conference on Recommender Systems(RecSys)
Neural Collaborative Filtering	2017	The Web Conference(WWW)
Deep Learning with Differential Privacy	2016	Computer and Communications Security(CCS)
Graph Attention Networks	2018	International Conference on Learning Representations(ICLR)
Modeling Relational Data with Graph Convolutional Networks	2018	European Semantic Web Conference(ESWC)
XGBoost: A Scalable Tree Boosting System	2016	Knowledge Discovery and Data Mining(KDD)
Structural Deep Network Embedding	2016	Knowledge Discovery and Data Mining(KDD)

Table S1-20. Leading article details

Leading article	Nodes	Edges
Inductive Representation Learning on Large Graphs	2071	11398
Wide & Deep Learning for Recommender Systems	1134	4563
Membership Inference Attacks Against Machine Learning Models	1064	6158
Deep Neural Networks for YouTube Recommendations	1161	3799
Neural Collaborative Filtering	1711	8466
Deep Learning with Differential Privacy	1548	8756
Graph Attention Networks	1101	3443
Modeling Relational Data with Graph Convolutional Networks	1103	3796
XGBoost: A Scalable Tree Boosting System	6884	12437
Structural Deep Network Embedding	1417	7747

Table S1-21. citation network overviews

S2 Model

S2.1 Idea tree extraction algorithm

There are too much redundant links in the citation network which has little academic influence on the citing papers. Therefore, repetitive, invalid inheritance relationships need to be removed to clearly and accurately reproduce the flow of the idea, which can be achieved by assessing the similarity between papers. Ideally, we assume that any child article in the network except the leading article is inspired by one of the most essential citation (the more similar the more important) so that we can get an idea tree that reveals the inheritance of ideas. In this way, we can characterize different evolution patterns of ideas through different idea tree structures. There are three steps to extracting the idea tree from the citation network. Initially, the nodes in the network are represented as vectors in a high-dimensional space. Then calculate the reduction index of the node, and measure the importance of the connection according to the difference in the reduction index. Finally, we get the idea tree by cutting the edges between the node pairs which have the largest reduction index difference.

The distance of academic articles in high-dimensional space

The first step is to measure the distance between the nodes by citation relationships. Particularly, we utilize graph embedding to measure such distance in high dimensional vector space. As for any target publication, we construct its citation network $G(V, E)$, among which the V represents the set of all the nodes and E represents the set of all the edges. Especially, $n = \|V\|$ is defined as the number of nodes in the network while $m = \|E\|$ is defined as the number of edges. A represents the adjacency matrix of the network, with the form of:

$$A = \begin{pmatrix} A_{11} & A_{12} & \dots & A_{1n} \\ A_{21} & A_{22} & \dots & A_{2n} \\ \vdots & \vdots & \ddots & \vdots \\ A_{n1} & A_{n2} & \dots & A_{nn} \end{pmatrix}$$

where $A_{ij} = 1$ represents that there exists reference relationship that paper v_i cites paper v_j. However, due to the existence of errors in the real data, $A_{ij} = 0$ doesn't absolutely means that paper v_i doesn't cite paper v_j. Actually, there seldom happens the phenomenon that two papers cite each other, which is determined by the reason that papers are usually published in sequential. Whereas, we do find few data in such form in the database, which we then turn such cite-each-other relationship to normal

reference relationship following the rule that the paper published later follows the paper published priorly.

Especially, we noticed that the leading paper cites none of the papers in the network since it's the earliest one, which blocks our later calculation for eigenvalues and eigenvectors. Considering this, we involve self-citation or self-loop to the leading paper, which allows subsequent eigenvalue decomposition. After the process above, we get W, the adjacency matrix with self-loop:

$$W = \begin{pmatrix} W_{11} & W_{12} & \dots & W_{1n} \\ W_{21} & W_{22} & \dots & W_{2n} \\ \vdots & \vdots & \ddots & \vdots \\ W_{n1} & W_{n2} & \dots & W_{nn} \end{pmatrix}$$

where $W_{ij} = A_{ij}$ when $v_i \neq v_j$, $W_{ij} = 0$ when $v_i = v_j$ and $\sum\limits_{j,j\neq i} A_{ij} > 0$, and $W_{ij} = 1$ when $v_i = v_j$ and $\sum\limits_{j,j\neq i} A_{ij} = 0$. Continue to process the adjacency matrix with self-loop W, we get the output matrix with self-loop D:

$$D = \begin{pmatrix} d_1 & 0 & \dots & 0 \\ 0 & d_2 & \dots & 0 \\ \vdots & \vdots & \ddots & \vdots \\ 0 & 0 & \dots & d_n \end{pmatrix}$$

which is a diagonal matrix with $d_i = \sum\limits_j W_{ij}$ and $d_i \neq 0$.

Considering that D is an diagonal matrix, we will easily to get $D^{-\frac{1}{2}}$. And based on this, we get the Laplace Matrix with self-loop L:

$$L = D - W$$

and normalized Laplace Matrix with self-loop:

$$L_{normal} = D^{-\frac{1}{2}}(D - W)D^{-\frac{1}{2}} \tag{S1}$$

with both matrix positive semidefinite.

Based on this, we then do eigenvalue decomposition on the normalized Laplace Matrix with self-loop, and acquire N eigenvalues and corresponding N eigenvectors. Generally Speaking, we will select the first k eigenvalues and the corresponding eigenvectors, which indicates that we promote the original citation network to the k-dimension space. To adequately exploit the data, we choose $k = n$ for later analysis, while k can be selected from 2 to n in real-world to reduce the calculation cost of huge amount of data. Then for any two papers v_i and v_j in citation network, the distance of them in k-dimensional space is $d_{ij} = \left\| eigvector_{v_i} - eigvector_{v_j} \right\|_2$, and we also get the distance matrix d of the papers in k-dimensional space:

$$d = \begin{pmatrix} d_{11} & d_{12} & \dots & d_{1n} \\ d_{21} & d_{22} & \dots & d_{2n} \\ \vdots & \vdots & \ddots & \vdots \\ d_{n1} & d_{n2} & \dots & d_{nn} \end{pmatrix}$$

Besides, we define $MaxDistance = \max_{i,j} \{d_{ij}A_{ij}\}$, which can be intuitively understand as the maximum distance in high-dimensional space for all edges existed in networks.

Based on the distance measurement in high-dimensional space, we exploit the random walk algorithm to calculate the reduction index of every node to the entire network. Whereas, before defining the reduction index of a specific node to the entire network, we need define the reduction index of single node to another (node pair) first.

The reduction index of one node to another (nodepair)

We first define the reduction index between nodepair. For nodepair (v_i, v_j), to measure the similarity of research content between v_i and v_j, we define the reduction index of nodepair as the sum of the weighted Dijkstra path [1] from v_i to all v_{j_k} in v_j's reference list, where the weight is the distance between two adjacent nodes on the path in the high-dimensional space.

$$ReductionIndex_{v_i,v_j} = \sum_{v_{j_k}} dijkstra_{v_i,v_{j_k}} \tag{S2}$$

Specially, for any v_{j_k},the weighted dijkstra path from v_i to v_{j_k} is weighted sum of edges in dijkstra path when there exists path from v_i to v_{j_k}, and $MaxDistance \times AverageStep$ when there exists no path from v_i to v_{j_k}, where $AverageStep$ is the average of the number of steps (edges) between every nodepair that can reach each other, whether in single step or multiple steps.

Similar to Symeonidis et al. [2], the weighted shortest path is introduced to calculate the similarity between non-neighboring nodes. We are also inspired by the idea that 'if two nodes are connected to a similar node, then two nodes are similar' [3].For article v_j, the articles in its reference list can be considered as the source of its ideas. Therefore it can be considered that the closer the article v_i is to the articles in the reference list of v_j, the more similar it is to the research content of article v_j.

Besides, for the two situations depending on whether there exists path from v_i to v_{j_k} or not, interpretations are as follows.

1. For the situation that there exists path from v_i to v_{j_k}, such dijkstra path should quantify the distance between v_i and v_{j_k}, whether they are connected directly or indirectly via paths.

2. For the situation that there exists no path from v_i to v_{j_k}, the distance calculated should be significantly larger than the distance when there exists path. Therefore, we use the $MaxDistance$ to make the value significantly large. And for the interpretation of the value of $MaxDistance$, we deem that the distance of a virtual edge (not really existing in the network) should be also significant larger than the distance of really existing edges. And for $AverageStep$, it can be interpreted that such $MaxDistance$ is for a direct virtual edge, while the virtual path from v_i to v_{j_k} may consists not only single virtual edge but multiple virtual edges that connected indirectly.

After defining the reduction index of single node to another (node pair), we will then define the reduction index of the entire network for any specific node.

The reduction index of any specific node to the entire network

For node v, its reduction index to the entire network G is defined as the sum of its reduction index to all other nodes in the network.

$$ReductionIndex_{v,G} = \sum_{u \in V/v} ReductionIndex_{v,u} \tag{S3}$$

Reduction index to the entire network helps us judge the importance of citations. The greater of the difference in the reduction index of two nodes to the network is, the less important the reference relationship between them is. Therefore, we find undirected loops and cut the unimportant nodepair according to the difference in reduction index to the entire network while maintaining the connectivity in Directed Graph conditions. During the process, two fundamental but significant principles should be followed:

1. Cut the nodepair with largest difference in reduction index to the entire network. For this principle, we sort the nodepairs according to the difference in reduction index to the entire network, and attempt to cut them in descending order. The specific criteria to whether cut it or not is stated in pricinple 2.

2. Maintain the connectivity in Directed Graph conditions
For this pricinple, we do not cut the edges that represents the last reference relationship still existing in the graph, and we will skip such edges when sorting and selecting the edges to cut. In such conditions, if every article except leading paper reserves only one reference relationship, then the output we get after cutting the edges will be undoubtedly a tree structure.

Following the two pricinples above, we do obtain an idea tree that reflects the idea flow of the citation network, which satisfies two properties:

1. The leading work is the only root node of the idea tree, while the whole structure is rooted on the leading node.

2. Starting from the root node of the idea tree, by doing inverse traversing operation of citation relationship, every node, or every paper in the idea tree, can be reached during the traversing.

S2.2 The calculation of knowledge entropy

Based on the idea tree, we can start from the structure of the tree and utilize structural information to measure the knowledge quality of academic articles. Specifically, the reason why we choose entropy to measure the quality of knowledge is that the entropy can measure the influence on uncertainty of the paper, which is compared with the situation that the paper does not exist. The uncertainty exists between two situations: With the paper involved, the structure of the idea tree is determined slightly; And without the paper involved, some structure of the idea tree is still unknown. Therefore, the larger a paper influences the idea tree, the structure of the idea tree is more certain, and the larger its knowledge entropy is.

Subtree entropy

For an academic paper a, the Subtree Entropy of a is defined as follows:

$$H\left(a\right) = -\frac{g_a}{2m}\log\left(\frac{V_a}{V_{a^-}}\right) \tag{S4}$$

The definition of subtree entropy follows the definition of structure entropy in [4], which measures the high-dimensional information embedded in network structures with the help of the partition tree. In the subtree entropy, g_a represents the number of the edges in the original citation network from the nodes in the subtree rooted on a in the idea tree to the nodes out of the subtree. m represents the number of edges in the idea tree. The larger the g_a, the more complex the structure associated with the subtree rooted on a. Therefore, the term $\frac{g_a}{2m}$ measures the importance of the subtree rooted on

a to the whole idea tree. V_a represents the number of the nodes in the subtree rooted on a while V_{a^-} represents the number of the nodes in the subtree rooted on a's parent node. The term $-\log\left(\frac{V_a}{V_{a^-}}\right)$ measures the uncertainty of the subtree rooted on a to its parent subtree. Generally speaking, the subtree entropy can measure the effect of the presence or absence of the corresponding subtree on the uncertainty of the whole idea tree. In this case, the greater the influence of a subtree on the idea tree, the greater the subtree entropy.

Mutual knowledge entropy and conditional knowledge entropy

With the definition of subtree entropy above, the definition of mutual knowledge entropy is also given as follows:

$$I(a,b) = -\frac{g_{ab}}{4m}\log\left(\frac{V_a V_b}{V_{ab^-}^2}\right) \tag{S5}$$

where g_{ab} represents the number of the edges in the original citation network from the nodes in the subtree rooted on a and the subtree rooted on b in the idea tree to the nodes out of the two subtrees. m represents the number of edges in the idea tree. V_a represents the number of the nodes in the subtree rooted on a, V_b represents the number of the nodes in the subtree rooted on b, while V_{ab^-} represents the number of the nodes in the subtree rooted on a and b's parent node, which indicates that a and b should have the same parent node, or the two nodes should locate in similar positions in the idea tree. The mutual knowledge entropy measures the degree of overlap of the knowledge contained in two subtrees. It can be considered that the overlapping knowledge is not created by these subtrees but inherited from the parent node.

Considering the definition form and the character of mutual knowledge entropy, it satisfies:

$$I(a,b) = I(b,a)$$

which reflects the symmetry of mutual knowledge entropy.

$$I(a,a) = H(a)$$

which reflects the self-symmetry of mutual knowledge entropy.

With the mutual knowledge entropy defined above, the conditional knowledge entropy is further defined:

$$H(a \mid b) = H(a) - I(a,b) = \frac{g_{ab}}{4m}\log\frac{V_a V_b}{V_{ab^-}^2} - \frac{g_a}{2m}\log\frac{V_a}{V_{a^-}}$$

And

$$H(a,b) = H(a) + H(b) - I(a,b) = -\frac{g_a - g_{ab}}{2m}\log\frac{V_a}{V_{a^-}} - \frac{g_b - g_{ab}}{2m}\log\frac{V_b}{V_{a^-}}$$

Knowledge entropy

Based on the subtree entropy and mutual knowledge entropy above, the definition of knowledge entropy is given as follows: at timestamp t, the citation network related to the target publication is $G^t = (V^t, E^t)$. The idea tree extracted from the network is $IdeaTree(G^t)$. For any paper v belong to $IdeaTree(G^t)$ except the leading work, we

define its knowledge entropy $KE^{t}\left(v\right)$ as:

$$KE^{t}\left(v\right) = H^{t}\left(v\right) - \sum_{v_i \in C^{t}(v)} H^{t}\left(v_i\right) + \sum_{v_i, v_j \in C^{t}(v), i \neq j} I^{t}\left(v_i, v_j\right) \tag{S6}$$

where $H^{t}\left(v\right)$ represents the subtree entropy of the subtree led by node v at t, $C^{t}\left(v\right)$ represents the children of v in $IdeaTree\left(G^{t}\right)$ at t, and $I^{t}\left(v_i, v_j\right)$ represents the mutual subtree entropy of the subtree led by v_i and v_j at t. Knowledge entropy is composed of two parts. The first part $H^{t}\left(v\right) - \sum_{v_i \in C^{t}(v)} H^{t}\left(v_i\right)$ is the subtree entropy of node v minus the subtree entropy of its child nodes, which quantifies the influence of node v itself on the formation of the idea tree structure by excluding the influence of child nodes $C^{t}\left(v\right)$. The first part may be negative, which indicates that the child nodes has more influence on the network structure than the parent node. The second part $\sum_{v_i, v_j \in C^{t}(v), i \neq j} I^{t}\left(v_i, v_j\right)$ reflects the amount of knowledge inherited by the child nodes $C^{t}\left(v\right)$ from the parent node v utilizing the mutual knowledge entropy from the side. Although the first part of KE may be negative when the knowledge of a parent node is inherited by a large number of child nodes, it causes the second term of the formula to increase, so we still consider it to have high knowledge quality. In the actual calculation, when the second term of the formula is very small, resulting in a negative value of KE, we directly set KE to 0 considering that the amount of knowledge of a scientific article cannot be negative. In this case, the article neither influences the structure sufficiently nor fails to create valuable knowledge to be inherited, so we consider it to have a low knowledge quality.

As for the leading article, since it has no parent node, its subtree entropy cannot be calculated directly. However, considering the numerical difference between subtree entropy and knowledge entropy, the influnence of subtree entropy on knowledge entropy can be ignored. Therefore, for the leading article v_s, its knowledge entropy is given as follows.

$$KE^{t}\left(v_s\right) = - \sum_{v_i \in C^{t}(v_s)} H^{t}\left(v_i\right) + \sum_{v_i, v_j \in C^{t}(v_s), i \neq j} I^{t}\left(v_i, v_j\right) \tag{S7}$$

where $C^{t}\left(v_s\right)$ represents the children node of leading article in $IdeaTree\left(G^{t}\right)$ at t.

S2.3 The fitting of idea limit formula

Assuming that the form of the idea limit formula is $\Delta D^{t}(v) = \log \frac{KE^{t}(v)}{(t-t_0)^{\gamma}}$, with the help of the least squares method, we use the evolution data of idea tree and knowledge entropy as the sample data to fit the time attenuation coefficient γ. For any high knowledge entropy node v in any idea tree, node v becomes visible at t_0, i.e. $KE^{t_0}\left(v\right) \geq M$, taking node v as a reference, the valid depth of the subtree led by node v is 0 at t_0. We know that the maximum valid depth $MaxVD_{subtree_v}$ of the subtree led by node v up to the current time t_{now} is $VD^{t_{now}}_{subtree_v}$, at any moment $\bar{t}$ between t_0 and t_{now}, we can get the sample data $\Delta D^{\bar{t}}(v)$ of $\Delta D^{t}(v)$: $\Delta D^{\bar{t}}(v) = MaxVD_{subtree_v} - VD^{\bar{t}}_{subtree_v}$. Similarly, we know that the knowledge entropy of node v at $\bar{t}$ is $KE^{\bar{t}}(v)$, therefore, we can get the sample data $(KE^{\bar{t}_i}(v_j), \bar{t}_i - t_0, \Delta D^{\bar{t}_i}(v_j))$ from all the idea trees to fit the formula. We transform the form of the formula to $\log KE^{t}(v) - \Delta D^{t}(v) = \gamma \log(t - t_0)$, and the value of a can be obtained according to the least squares method:

$$\hat{\gamma} = \arg\min_{\gamma} \sum_{i,j} ((\log KE^{\bar{t}_i}(v_j) - \Delta D^{\bar{t}_i}(v_j)) - \gamma \log(\bar{t}_i - t_0))^2 \tag{S8}$$

Let the derivative of the objective function to γ be 0, we can get $\hat{\gamma} = \frac{\sum_{i,j}(\log KE^{\bar{t}_i}(v_j) - \Delta D^{\bar{t}_i}(v_j))}{\sum_{i,j}\log(\bar{t}_i - t_0)}$, and the fitting result of γ is 1.914.

S3 Examples of citation networks pioneered by high-impact publications

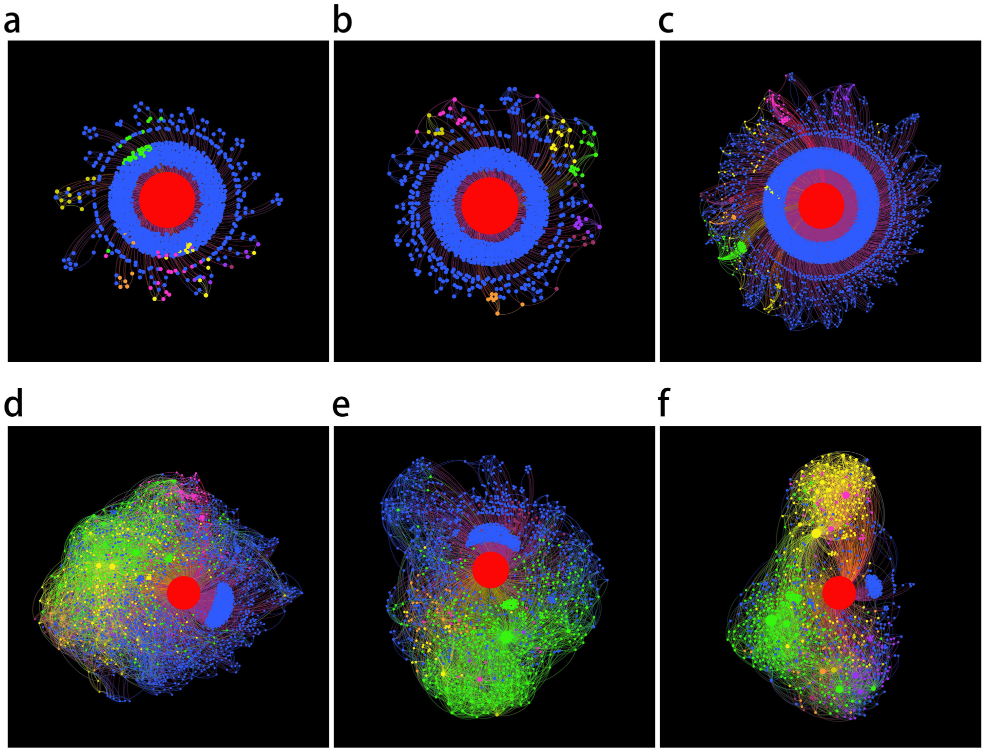

Fig S3-1. Examples of citation networks pioneered by high-impact publications in 2021. (a-f) The citation networks led by 'Applied Predictive Modeling', 'A survey of deep neural network architectures and their applications', 'PyTorch: An Imperative Style, High-Performance Deep Learning Library', 'Improved Training of Wasserstein GANs', 'FlowNet 2.0: Evolution of Optical Flow Estimation with Deep Networks' and 'Context Encoders: Feature Learning by Inpainting'. The red node in the network is the leading article. Except for the blue nodes, other nodes with the same color represent that they belong to a larger community, and the blue node does not belong to these communities. The size of the node is positively related to its citation within the network. Different types of leading articles' ideas will make the citing papers associated in different ways.

As shown in Fig. S3-1(a-c), these networks are led by textbook, survey and software toolkit, respectively. All these networks only form a uniform ring around the summative leading article. As shown in Fig. S3-1(d-f), these networks are led by innovative research articles. 'Improved Training of Wasserstein GANs' proposed a new method to improve the stability of GAN training; 'FlowNet 2.0: Evolution of Optical Flow Estimation with Deep Networks' is a classic article on the estimation of optical flow using deep learning; 'Context Encoders: Feature Learning by Inpainting' is A is the first article to use deep learning to solve image inpainting problems. All networks led by these three articles form more complex structures.

S4 Examples of six evolution patterns

S4.1 Pattern 1: The VD of summative work is hard to exceed one

Principal component analysis: a review and recent developments

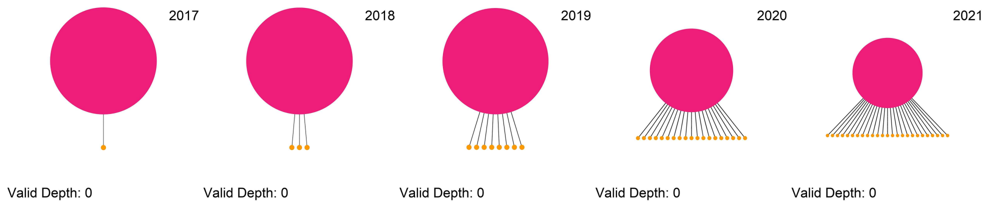

Fig S4-1. The evolution of idea tree structures led by 'Principal component analysis: a review and recent developments'

The leading work of the idea tree is 'Principal component analysis: a review and recent developments'. It was published in 2016, and it has already attracted 1,203 citations until 2021. leading work tends to summarize existing knowledge, so it is not very inspiring for child nodes and cannot provide new research idea. By observing the evolution of the idea trees over time, we find that even though the citation continues to increase, it never breeds new high-impact nodes within it, which stagnates its VD at zero.

Multilevel Analysis : Techniques and Applications, Third Edition

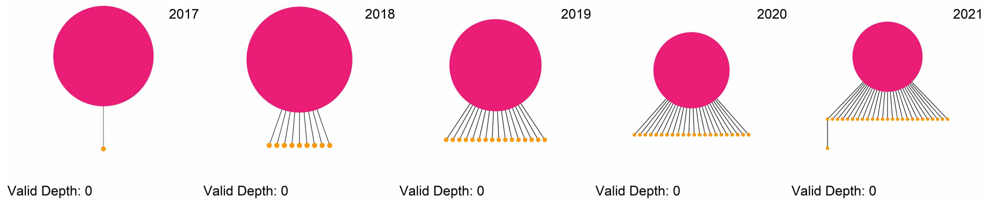

Fig S4-2. The evolution of idea tree structures led by 'Multilevel Analysis : Techniques and Applications, Third Edition'

The leading work of the idea tree is 'Multilevel Analysis : Techniques and Applications, Third Edition'. It was published in 2017, and it has already attracted 2,321 citations until 2021. leading work tends to summarize existing knowledge, so it is not very inspiring for child nodes and cannot provide new research idea. By observing the evolution of the idea trees over time, we find that even though the citation of target publication continues to increase, it never breeds new high-impact nodes within it, which stagnates its VD at zero.

An Introduction to Medical Statistics

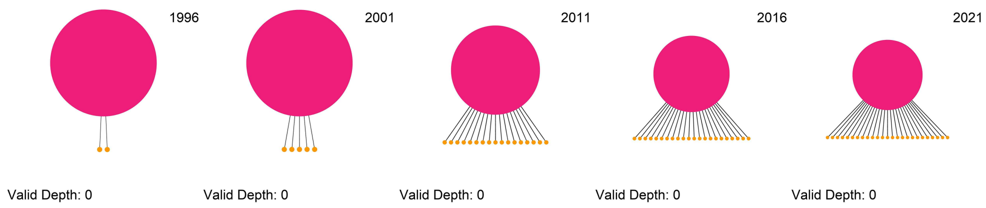

Fig S4-3. The evolution of idea tree structures led by 'An Introduction to Medical Statistics'

The leading work of the idea tree is 'An Introduction to Medical Statistics'. It was published in 1987, and it has already attracted 2,128 citations until 2021. leading work tends to summarize existing knowledge, so it is not very inspiring for child nodes and cannot provide new research idea. By observing the evolution of the idea trees over time, we find that even though the citation of target publication continues to increase, it never breeds new high-impact nodes within it, which stagnates its VD at zero.

TensorFlow: a system for large-scale machine learning

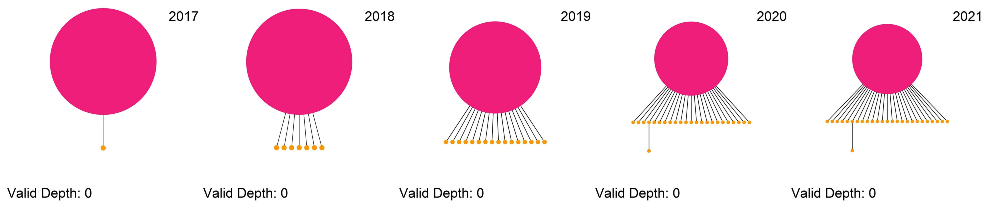

Fig S4-4. The evolution of idea tree structures led by 'TensorFlow: a system for large-scale machine learning'

The leading work of the idea tree is 'TensorFlow: a system for large-scale machine learning'. It was published in 2016, and it has already attracted 5,199 citations until 2021. leading work tends to summarize existing knowledge, so it is not very inspiring for child nodes and cannot provide new research idea. By observing the evolution of the idea trees over time, we find that even though the citation of target publication continues to increase, it never breeds new high-impact nodes within it, which stagnates its VD at zero.

S4.2 Pattern 2: The increase in VD needs to be driven by non-trivial child nodes

Recent Arctic amplification and extreme mid-latitude weather

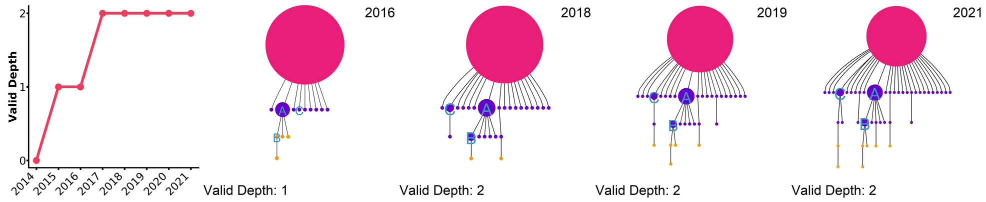

Fig S4-5. The evolution of idea tree structures led by 'Recent Arctic amplification and extreme mid-latitude weather'

Label	Title	KE	Year
leading article	Recent Arctic amplification and extreme mid-latitude weather	4926.7914	2014
A	Robust Arctic sea-ice influence on the frequent Eurasian cold winters in past decades	203.0495	2014
B	Two Distinct Influences of Arctic Warming on Cold Winters over North America and East Asia	45.0308	2015
C	The impact of Arctic warming on the mid-latitude jet-stream: Can it? Has it? Will it?: Impact of Arctic warming on the mid-latitude jet-stream	38.9499	2015

Table S4-1. Child articles details

The pioneering work of the idea tree is 'Recent Arctic amplification and extreme mid-latitude weather'. It was published in 2014, and it has already attracted 1,036 citations until 2021. Child node A attracts much external attention, making the subtree led by it flourish and causing new high KE node B to be born under its subtree, which increases the VD to two. This shows that paper A creates valuable knowledge and drives the target publication's idea development.

Processing seismic ambient noise data to obtain reliable broad-band surface wave dispersion measurements

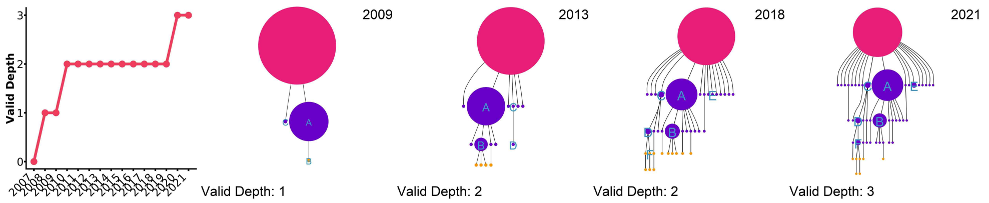

Fig S4-6. The evolution of idea tree structures led by 'Processing seismic ambient noise data to obtain reliable broad-band surface wave dispersion measurements'

Label	Title	KE	Year
leading article	Processing seismic ambient noise data to obtain reliable broad-band surface wave dispersion measurements	4240.8127	2007
A	Ambient noise Rayleigh wave tomography of New Zealand	649.6994	2007
B	Surface wave tomography of the western United States from ambient seismic noise: Rayleigh and Love wave phase velocity maps	229.0305	2008
C	Earthquake ground motion prediction using the ambient seismic field	23.1898	2008
D	Testing Community Velocity Models for Southern California Using the Ambient Seismic Field	22.5306	2008
E	Using instantaneous phase coherence for signal extraction from ambient noise data at a local to a global scale	12.5919	2011
F	Tutorial on seismic interferometry: Part 1 — Basic principles and applications	15.2850	2010

Table S4-2. Child articles details

The pioneering work of the idea tree is 'Processing seismic ambient noise data to obtain reliable broad-band surface wave dispersion measurements'. It was published in 2007, and it has already attracted 1,307 citations until 2021. Child node A attracts much external attention, making the subtree led by it flourish and causing new high KE node B to be born under its subtree, which increases the VD to three. This shows that paper A creates valuable knowledge and drives the target publication's idea development.

Overcoming catastrophic forgetting in neural networks

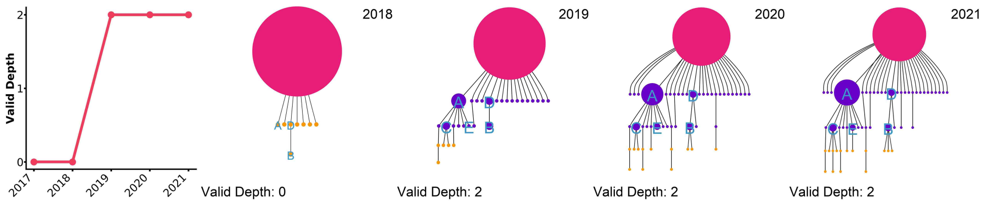

Fig S4-7. The evolution of idea tree structures led by 'Overcoming catastrophic forgetting in neural networks'

Label	Title	KE	Year
leading article	Overcoming catastrophic forgetting in neural networks	6147.1529	2017
A	iCaRL: Incremental Classifier and Representation Learning	466.7194	2017
B	Continual learning through synaptic intelligence	47.7389	2017
C	Encoder Based Lifelong Learning	59.0671	2017
D	Learning without Forgetting	38.0666	2018
E	Continual Lifelong Learning with Neural Networks: A Review	23.7898	2019

Table S4-3. Child articles details

The pioneering work of the idea tree is ‘Overcoming catastrophic forgetting in neural networks’. It was published in 2017, and it has already attracted 1,439 citations until 2021. Child node A attracts much external attention, making the subtree led by it flourish and causing two new high KE node C, E to be born under its subtree, which increases the VD to two. This shows that paper A creates valuable knowledge and drives the target publication’s idea development.

Globally and locally consistent image completion

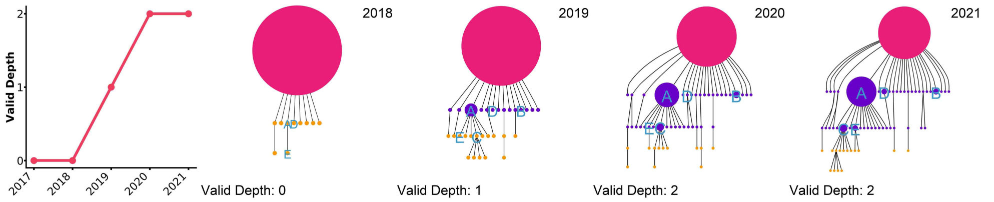

Fig S4-8. The evolution of idea tree structures led by ‘Globally and locally consistent image completion’

Label	Title	KE	Year
leading article	Globally and locally consistent image completion	2992.0601	2017
A	Generative Image Inpainting with Contextual Attention	567.8963	2018
B	Generative Image Inpainting with Contextual Attention (arXiv)	16.9506	2018
C	Free-Form Image Inpainting With Gated Convolution	64.8233	2018
D	High-Resolution Image Synthesis and Semantic Manipulation with Conditional GANs	10.2987	2018
E	Image Inpainting for Irregular Holes Using Partial Convolutions	11.9479	2018

Table S4-4. Child articles details

The pioneering work of the idea tree is ‘Globally and locally consistent image completion’. It was published in 2017, and it has already attracted 1,046 citations until

2021. Child node A attracts much external attention, making the subtree led by it flourish and causing new high KE node C, E to be born under its subtree, which increases the VD to two. This shows that paper A creates valuable knowledge and drives the target publication's idea development.

S4.3 Pattern 3: The continuous increase of the VD needs to be stimulated by the influence relay of multiple high KE nodes

Improved techniques for training GANs

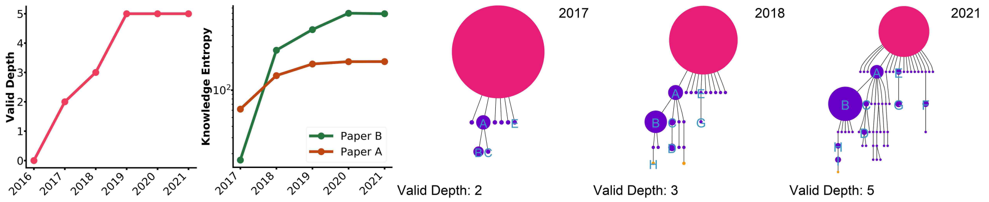

Fig S4-9. The evolution of idea tree structures led by 'Improved techniques for training GANs'

Label	Title	KE	Year
leading article	Improved techniques for training GANs	5339.2979	2016
A	Energy-based Generative Adversarial Networks	206.1023	2017
B	Image-to-Image Translation with Conditional Adversarial Networks	692.8008	2017
C	Calibrating Energy-based Generative Adversarial Networks	13.0860	2017
D	Improved Training of Wasserstein GANs (arXiv)	22.7750	2017
E	Learning from Simulated and Unsupervised Images through Adversarial Training	23.9048	2017
F	Improved Training of Wasserstein GANs	34.6206	2017
G	Unpaired Image-to-Image Translation Using Cycle-Consistent Adversarial Networks	41.4849	2017
H	StackGAN: Text to Photo-Realistic Image Synthesis with Stacked Generative Adversarial Networks	17.4041	2017
I	Learning to Discover Cross-Domain Relations with Generative Adversarial Networks	25.9975	2017

Table S4-5. Child articles details

The pioneering work of the idea tree is 'Improved techniques for training GANs'. It was published in 2016, and it has already attracted 2,247 citations until 2021. The KE of child node A is the first to highlight, but after 2018, its KE almost stopped increasing. At this time, the KE of the child node B, which was directly inspired by paper A, has begun to emerge and exceed paper A. Paper B took over the task of motivating the VD increase. The idea tree achieves multiple inheritances of ideas and ensures that it can continue to attract attention. Several child nodes with high KE were birthed under the subtree led by paper B, and made the VD continuously increased to five.

Matching networks for one shot learning

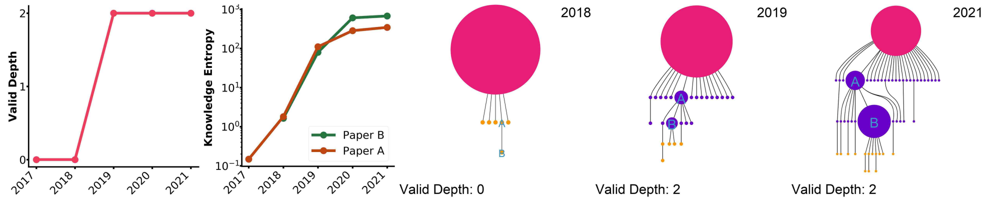

Fig S4-10. The evolution of idea tree structures led by 'Matching networks for one shot learning'

Label	Title	KE	Year
leading article	Matching networks for one shot learning	6619.5201	2016
A	Meta Networks	345.9163	2017
B	Learning to Compare: Relation Network for Few-Shot Learning	677.4604	2018

Table S4-6. Child articles details

The pioneering work of the idea tree is 'Matching networks for one shot learning'. It was published in 2016, and it has already attracted 1,515 citations until 2021. The KE of child node A is the first to highlight, but after 2019, its KE almost stopped increasing. At this time, the KE of the child node B, which was directly inspired by paper A, has begun to emerge and exceed paper A. Paper B took over the task of motivating the VD increase. The idea tree achieves multiple inheritances of ideas from the leading article and ensures that it can continue to attract attention. This makes the VD continuously increased to two.

A Meta-Analysis of Global Urban Land Expansion

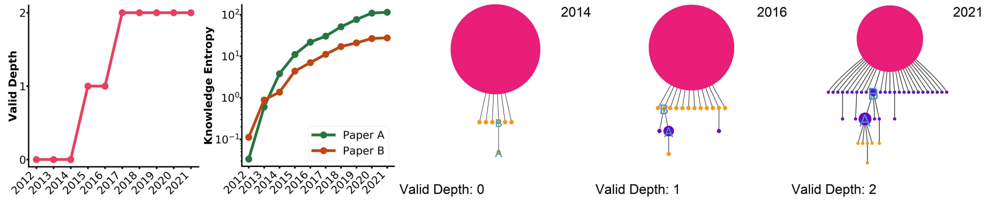

Fig S4-11. The evolution of idea tree structures led by 'A Meta-Analysis of Global Urban Land Expansion'

Label	Title	KE	Year
leading article	Matching networks for one shot learning	2673.4137	2011
A	Global forecasts of urban expansion to 2030 and direct impacts on biodiversity and carbon pools	114.8178	2012
B	Urban land teleconnections and sustainability	27.6795	2012

Table S4-7. Child articles details

The pioneering work of the idea tree is 'A Meta-Analysis of Global Urban Land Expansion'. It was published in 2011, and it has already attracted 1,118 citations until 2021. The KE of child node B is the first to highlight, but after 2013, The growth of its KE started to slow down. At this time, the KE of the child node A, which was directly inspired by paper B, has begun to emerge and exceed paper B. Paper A took over the task of motivating the VD increase. The idea tree achieves multiple inheritances of ideas from the leading article and ensures that it can continue to attract attention. This mades the VD continuously increased to two.

Cleavage of GSDMD by inflammatory caspases determines pyroptotic cell death

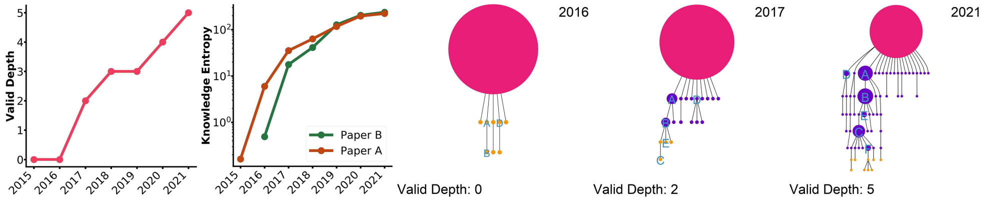

Fig S4-12. The evolution of idea tree structures led by 'Cleavage of GSDMD by inflammatory caspases determines pyroptotic cell death'

Label	Title	KE	Year
leading article	Cleavage of GSDMD by inflammatory caspases determines pyroptotic cell death	2864.9248	2015
A	Gasdermin D is an executor of pyroptosis and required for interleukin-1β secretion	223.7697	2015
B	Inflammasome-activated gasdermin D causes pyroptosis by forming membrane pores	238.3985	2016
C	Chemotherapy drugs induce pyroptosis through caspase-3 cleavage of a gasdermin	167.2502	2017
D	A Genome-wide CRISPR (Clustered Regularly Interspaced Short Palindromic Repeats) Screen Identifies NEK7 as an Essential Component of NLRP3 Inflammasome Activation	14.3097	2016
E	Pyroptosis is driven by non-selective gasdermin-D pore and its morphology is different from MLKL channel-mediated necroptosis.	11.3740	2016
F	Gasdermins: Effectors of Pyroptosis	18.3531	2017

Table S4-8. Child articles details

The pioneering work of the idea tree is 'Cleavage of GSDMD by inflammatory caspases determines pyroptotic cell death'. It was published in 2015, and it has already attracted 1,583 citations until 2021. The KE of child node A is the first to highlight, but after 2019, the KE of the child node B, which was directly inspired by paper A, has begun to exceed paper A. Paper B took over the task of motivating the VD increase. The idea tree achieves multiple inheritances of ideas and ensures that it can continue to attract

attention. Several child nodes with high KE were birthed under the subtree led by paper B, and made the VD continuously increased to five.

S4.4 Pattern 4: The presence of overpowered child nodes can ruin the increase in the VD

The Kinetics Human Action Video Dataset

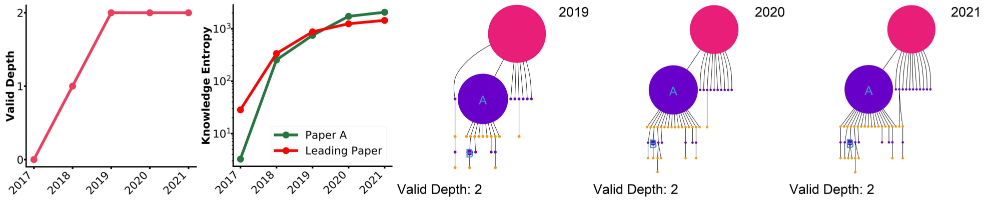

Fig S4-13. The evolution of idea tree structures led by 'The Kinetics Human Action Video Dataset'

Label	Title	KE	Year
leading article	The Kinetics Human Action Video Dataset	1447.5796	2017
A	Quo Vadis, Action Recognition? A New Model and the Kinetics Dataset	2070.2597	2017
B	Can Spatiotemporal 3D CNNs Retrace the History of 2D CNNs and ImageNet	31.6628	2018

Table S4-9. Child articles details

The pioneering work of the idea tree is 'The Kinetics Human Action Video Dataset'. It was published in 2017, and it has already attracted 1,043 citations until 2021. The KE of the child article A increases rapidly and approaches the order of magnitude of the leading article's KE. However, in the subtree led by article A, new high KE nodes are difficult to appear in large numbers. This indicates that the target publication's idea lost attention of new valuable knowledge and fell into a stagnation of development.

Estimating Corn Leaf Chlorophyll Concentration from Leaf and Canopy Reflectance

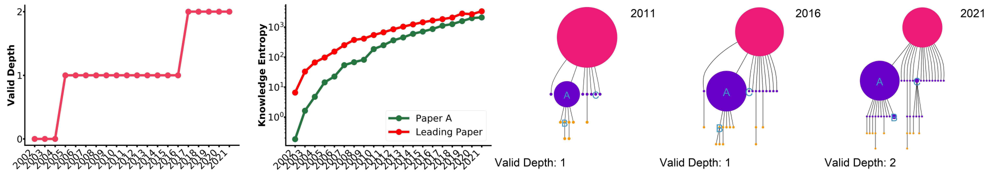

Fig S4-14. The evolution of idea tree structures led by 'Estimating Corn Leaf Chlorophyll Concentration from Leaf and Canopy Reflectance'

Label	Title	KE	Year
leading article	Estimating Corn Leaf Chlorophyll Concentration from Leaf and Canopy Reflectance	3378.7116	2000
A	Integrated narrow-band vegetation indices for prediction of crop chlorophyll content for application to precision agriculture	2099.8930	2002
B	Hyperspectral vegetation indices and novel algorithms for predicting green LAI of crop canopies: Modeling and validation in the context of precision agriculture	39.8294	2004
C	Wide Dynamic Range Vegetation Index for Remote Quantification of Biophysical Characteristics of Vegetation	14.7088	2004

Table S4-10. Child articles details

The pioneering work of the idea tree is ‘Estimating Corn Leaf Chlorophyll Concentration from Leaf and Canopy Reflectance’. It was published in 2000, and it has already attracted 1,281 citations until 2021. The KE of the child article A exceeds the leading article’s KE. However, in the subtree led by article A, new high KE nodes are difficult to appear in large numbers. This indicates that the target publication’s idea lost attention of new valuable knowledge and fell into a stagnation of development.

Thermal remote sensing of urban climates

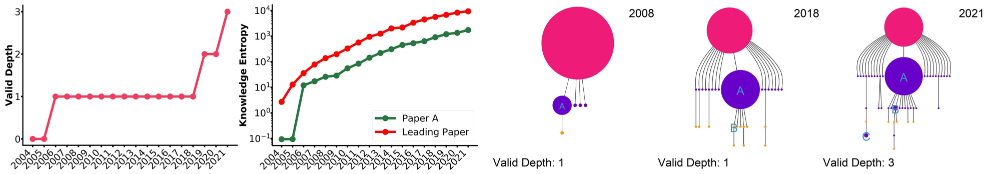

Fig S4-15. The evolution of idea tree structures led by ‘Thermal remote sensing of urban climates’

Label	Title	KE	Year
leading article	Thermal remote sensing of urban climates	9332.8724	2003
A	Estimation of land surface temperature-vegetation abundance relationship for urban heat island studies	1720.8779	2004
B	Surface Urban Heat Island Across 419 Global Big Cities	30.1179	2012
C	Satellite remote sensing of surface urban heat islands: Progress, challenges, and perspectives	14.3952	2018

Table S4-11. Child articles details

The pioneering work of the idea tree is ‘Thermal remote sensing of urban climates’. It was published in 2003, and it has already attracted 1,539 citations until 2021. The KE of the child article A increases rapidly and approaches the order of magnitude of the leading article’s KE. However, in the subtree led by article A, new high KE nodes are difficult to appear in large numbers. This indicates that the target publication’s idea lost attention of new valuable knowledge and fell into a stagnation of development.

Weyl Semimetal Phase in Noncentrosymmetric Transition-Metal Monophosphides

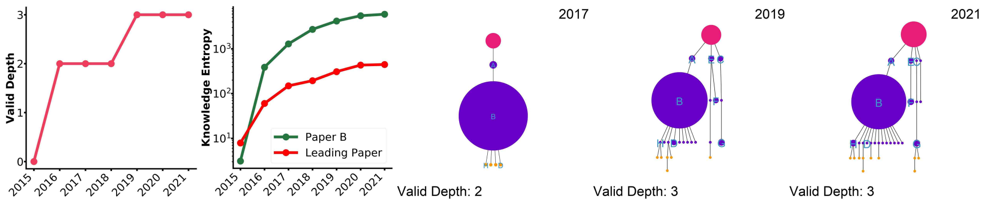

Fig S4-16. The evolution of idea tree structures led by 'Weyl Semimetal Phase in Noncentrosymmetric Transition-Metal Monophosphides'

Label	Title	KE	Year
leading article	Weyl Semimetal Phase in Noncentrosymmetric Transition-Metal Monophosphides	446.7779	2015
A	Experimental observation of Weyl points	21.0205	2015
B	Experimental discovery of Weyl semimetal TaAs	1085.0453	2015
C	Extremely large magnetoresistance and ultrahigh mobility in the topological Weyl semimetal candidate NbP	9.8317	2015
D	Observation of Weyl nodes in TaAs	7.5791	2015
E	Line-Node Dirac Semimetal and Topological Insulating Phase in Noncentrosymmetric Pnictides CaAgX (X = P, As)	13.3865	2016
F	Topological nodal line semimetals	28.1282	2016
G	Weyl and Dirac semimetals in three-dimensional solids	32.1386	2018
H	Triple Point Topological Metals	27.6119	2016

Table S4-12. Child articles details

The pioneering work of the idea tree is 'Weyl Semimetal Phase in Noncentrosymmetric Transition-Metal Monophosphides'. It was published in 2015, and it has already attracted 1,105 citations until 2021. The KE of the child article B increases rapidly and exceeds the leading article's KE. However, in the subtree led by article B, new high KE nodes are difficult to appear in large numbers. This indicates that the target publication's idea lost attention of new valuable knowledge and fell into a stagnation of development.

S4.5 Pattern 5: Stronger branches inhibit the increase in VD of weaker branches

Non-ideal interactions in calcic amphiboles and their bearing on amphibole-plagioclase thermometry

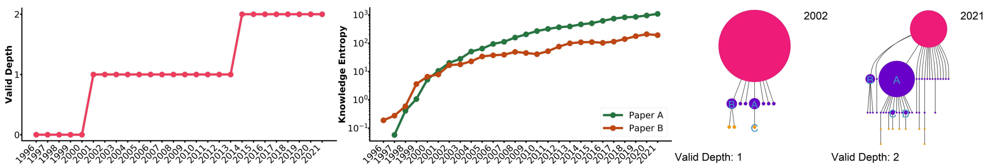

Fig S4-17. The evolution of idea tree structures led by ‘Non-ideal interactions in calcic amphiboles and their bearing on amphibole-plagioclase thermometry’

Label	Title	KE	Year
leading article	Non-ideal interactions in calcic amphiboles and their bearing on amphibole-plagioclase thermometry	2747.0623	1994
A	Nomenclature of amphiboles; Report of the Subcommittee on Amphiboles of the International Mineralogical Association, Commission on New Minerals and Mineral Names	1044.7652	1997
B	The effects of temperature and f O2 on the Al-in-hornblende barometer	192.8548	1995
C	Experimental phase-equilibrium study of Al- and Ti-contents of calcic amphibole in MORB; a semiquantitative thermobarometer	28.5731	1998
D	Temperature-induced Al-zoning in hornblendes of the Fish Canyon magma, Colorado	10.9639	2002

Table S4-13. Child articles details

The pioneering work of the idea tree is ‘Non-ideal interactions in calcic amphiboles and their bearing on amphibole-plagioclase thermometry’. It was published in 1994, and it has already attracted 1,612 citations until 2021. In the next layer of leading work, two nodes with high KE were born. In the early stage of the idea tree’s development, the subtree led by paper B first became prosperous. When the KE of paper A exceeds paper B, the subtree led by paper A began to become prosperous, and new high KE nodes were born in it, thus increasing the VD by one. In this process, the newly emerged branches attracted the attention of the outside world. In contrast, the subtree led by paper B began to be ignored, thus missing the golden opportunity for development, leading to stagnation of its development.

A dynamic global vegetation model for studies of the coupled atmosphere-biosphere system

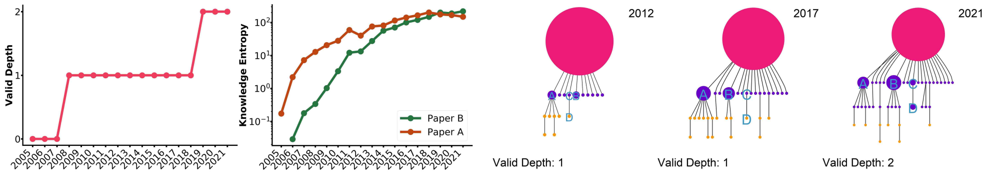

Fig S4-18. The evolution of idea tree structures led by 'A dynamic global vegetation model for studies of the coupled atmosphere-biosphere system'

Label	Title	KE	Year
leading article	A dynamic global vegetation model for studies of the coupled atmosphere-biosphere system	4525.8388	2005
A	Europe-wide reduction in primary productivity caused by the heat and drought in 2003	150.1318	2005
B	Climate-carbon cycle feedback analysis: Results from the C4MIP model intercomparison	220.0082	2006
C	The LMDZ4 general circulation model: climate performance and sensitivity to parametrized physics with emphasis on tropical convection	12.8155	2006
D	Key features of the IPSL ocean atmosphere model and its sensitivity to atmospheric resolution	12.4421	2010

Table S4-14. Child articles details

The pioneering work of the idea tree is 'A dynamic global vegetation model for studies of the coupled atmosphere-biosphere system'. It was published in 2005, and it has already attracted 1,467 citations until 2021. In the next layer of leading work, two nodes with high KE were born. In the early stage of the idea tree's development, the subtree led by paper A first became prosperous. When the KE of paper B exceeds paper A, the subtree led by paper B began to become prosperous. In contrast, the subtree led by paper A began to be ignored, thus missing the golden opportunity for development, leading to stagnation of its development.

Robust Responses of the Hydrological Cycle to Global Warming

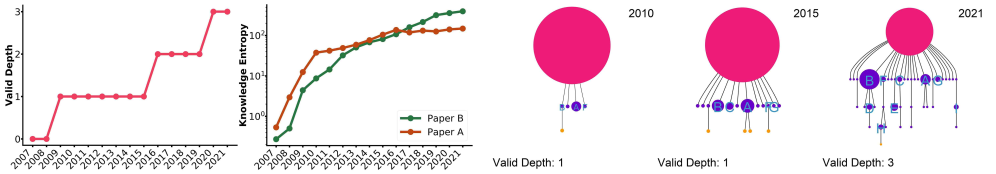

Fig S4-19. The evolution of idea tree structures led by 'Robust Responses of the Hydrological Cycle to Global Warming'

Label	Title	KE	Year
leading article	Robust Responses of the Hydrological Cycle to Global Warming	14496.8490	2006
A	How Much More Rain Will Global Warming Bring	148.7734	2007
B	Expansion of the Hadley cell under global warming	398.8780	2007
C	Mechanisms for the land/sea warming contrast exhibited by simulations of climate change	11.2109	2008
D	Controls of Global-Mean Precipitation Increases in Global Warming GCM Experiments	23.3428	2008
E	Changes in precipitation with climate change	81.6026	2011
F	Increased tropical Atlantic wind shear in model projections of global warming	15.3969	2007
G	The impact of global warming on the tropical Pacific Ocean and El Niño.	17.0690	2010
H	Effects of increased CO2 levels on monsoons	13.1628	2011
I	Increase in hourly precipitation extremes beyond expectations from temperature changes	10.2316	2008

Table S4-15. Child articles details

The pioneering work of the idea tree is 'Robust Responses of the Hydrological Cycle to Global Warming'. It was published in 2006, and it has already attracted 3,083 citations until 2021. In the next layer of leading work, two nodes with high KE were born. In the early stage of the idea tree's development, the subtree led by paper A first became prosperous. When the KE of paper B exceeds paper A, the subtree led by paper B began to become prosperous, and new high KE nodes were born in it, thus increasing the VD by one. In this process, the newly emerged branches attracted the attention of the outside world. In contrast, the branches led by paper A were ignored, thus missing the golden opportunity for development, leading to stagnation of its development.

Prototypical Networks for Few-shot Learning

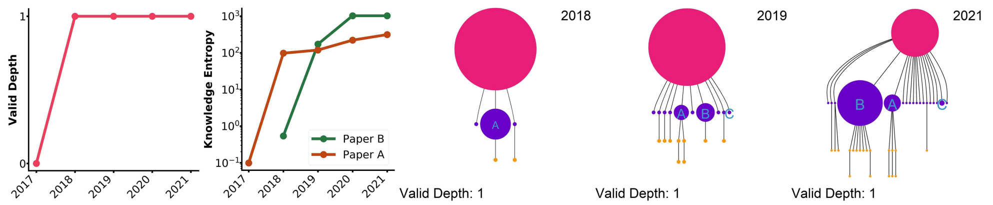

Fig S4-20. The evolution of idea tree structures led by 'Prototypical Networks for Few-shot Learning'

Label	Title	KE	Year
leading article	Prototypical Networks for Few-shot Learning	7627.8892	2017
A	Model-agnostic meta-learning for fast adaptation of deep networks	314.6073	2017
B	Learning to Compare: Relation Network for Few-Shot Learning	1027.7582	2018
C	Low-Shot Learning from Imaginary Data	17.4369	2018

Table S4-16. Child articles details

The pioneering work of the idea tree is ‘Prototypical Networks for Few-shot Learning’. It was published in 2017, and it has already attracted 1,964 citations until 2021. In the next layer of leading work, two nodes with high KE were born. In the early stage of the idea tree’s development, the subtree led by paper A first became prosperous. When the KE of paper B exceeds paper A, the subtree led by paper B began to become prosperous. In contrast, the subtree led by paper A began to be ignored, thus missing the golden opportunity for development, leading to stagnation of its development.

S4.6 Pattern 6: VD near the upper bound of development requires a large number of high KE nodes to drive

Unsupervised Representation Learning with Deep Convolutional Generative Adversarial Networks

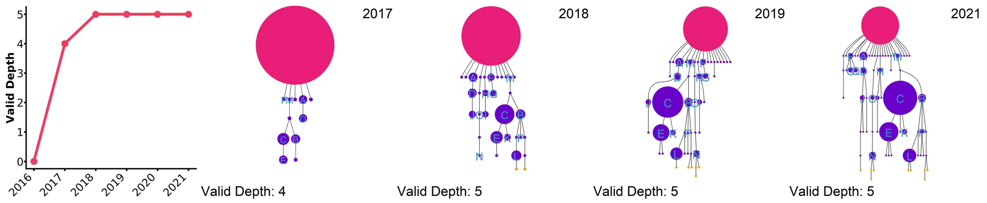

Fig S4-21. The evolution of idea tree structures led by ‘Unsupervised Representation Learning with Deep Convolutional Generative Adversarial Networks’

Label	Title	KE	Year
leading article	Unsupervised Representation Learning with Deep Convolutional Generative Adversarial Networks	21565.4562	2016
A	InfoGAN: interpretable representation learning by information maximizing generative adversarial nets	141.9261	2016
B	Improved techniques for training GANs	134.6050	2016
C	Improved techniques for training GANs	978.0934	2016
D	Conditional Image Synthesis With Auxiliary Classifier GANs	84.4415	2017
E	Image-to-Image Translation with Conditional Adversarial Networks	498.4536	2017
F	Attribute2Image: Conditional Image Generation from Visual Attributes	53.6515	2016
G	Coupled generative adversarial networks	29.1537	2016
H	Perceptual Losses for Real-Time Style Transfer and Super-Resolution	22.8999	2016
I	Generative Visual Manipulation on the Natural Image Manifold	18.2762	2016
J	Unrolled Generative Adversarial Networks	44.2670	2016
K	Unpaired Image-to-Image Translation Using Cycle-Consistent Adversarial Networks	46.5298	2017
L	Wasserstein GAN	316.0971	2017
M	Attribute2Image: Conditional Image Generation from Visual Attributes	16.5627	2016
N	Improved Training of Wasserstein GANs	114.3471	2017
O	Deconvolution and Checkerboard Artifacts	11.7450	2016
P	f -GAN: training generative neural samplers using variational divergence minimization	14.1597	2016
Q	Semantic Image Inpainting with Deep Generative Models	11.1524	2017

Table S4-17. Child articles details

The pioneering work of the idea tree is 'Unsupervised Representation Learning with Deep Convolutional Generative Adversarial Networks'. It was published in 2016, and it has already attracted 5,789 citations until 2021. A large number of high KE nodes are bred inside the idea tree, which makes the VD reach five.

Feature Pyramid Networks for Object Detection

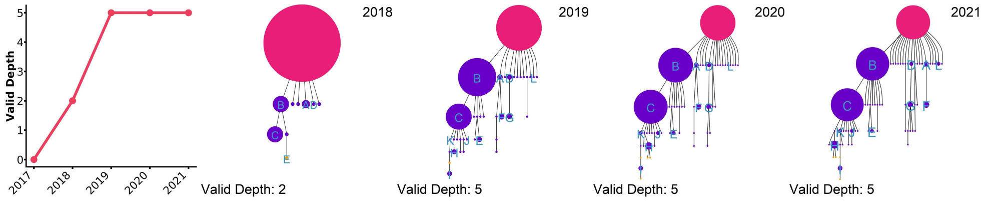

Fig S4-22. The evolution of idea tree structures led by 'Feature Pyramid Networks for Object Detection'

Label	Title	KE	Year
leading article	Feature Pyramid Networks for Object Detection	15048.9172	2017
A	Focal Loss for Dense Object Detection	59.7089	2017
B	Mask R-CNN	2412.6037	2017
C	Focal Loss for Dense Object Detection	1296.1841	2017
D	Speed/Accuracy Trade-Offs for Modern Convolutional Object Detectors	22.3072	2017
E	Mask R-CNN	19.9824	2017
F	YOLOv3: An Incremental Improvement	98.4034	2018
G	Cascade R-CNN: Delving Into High Quality Object Detection	70.0600	2018
H	CornerNet: Detecting Objects as Paired Keypoints	164.2707	2018
I	Encoder-Decoder with Atrous Separable Convolution for Semantic Image Segmentation	52.5990	2018
J	Path Aggregation Network for Instance Segmentation	12.4741	2018
K	Learning Transferable Architectures for Scalable Image Recognition	21.7402	2018
L	Frustum PointNets for 3D Object Detection from RGB-D Data	12.8733	2018

Table S4-18. Child articles details

The pioneering work of the idea tree is 'Feature Pyramid Networks for Object Detection'. It was published in 2017, and it has already attracted 5,031 citations until 2021. A large number of high KE nodes are bred inside the idea tree, which makes the VD reach five.

Inception-v4, Inception-ResNet and the Impact of Residual Connections on Learning

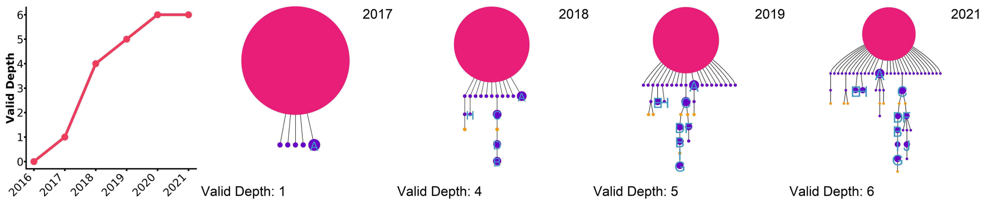

Fig S4-23. The evolution of idea tree structures led by 'Inception-v4, Inception-ResNet and the Impact of Residual Connections on Learning'

Label	Title	KE	Year
leading article	Inception-v4, Inception-ResNet and the Impact of Residual Connections on Learning	11336.1737	2016
A	Identity Mappings in Deep Residual Networks	96.9002	2016
B	Aggregated Residual Transformations for Deep Neural Networks	20.0488	2016
C	Speed/Accuracy Trade-Offs for Modern Convolutional Object Detectors	67.4979	2017
D	Mask R-CNN	25.2243	2017
E	MobileNets: Efficient Convolutional Neural Networks for Mobile Vision Applications	16.8268	2017
F	Mask R-CNN	30.6026	2017
G	Learning Transferable Architectures for Scalable Image Recognition	36.2898	2018
H	Xception: Deep Learning with Depthwise Separable Convolutions	23.0431	2017
I	Focal Loss for Dense Object Detection	13.8385	2017
J	Squeeze-and-Excitation Networks	26.8498	2018

Table S4-19. Child articles details

The pioneering work of the idea tree is 'Inception-v4, Inception-ResNet and the Impact of Residual Connections on Learning'. It was published in 2016, and it has already attracted 3,024 citations until 2021. A large number of high KE nodes are bred inside the idea tree, which makes the VD reach six.

High Serum IgG4 Concentrations in Patients with Sclerosing Pancreatitis

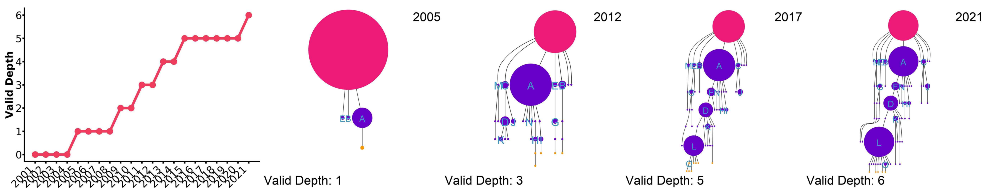

Fig S4-24. The evolution of idea tree structures led by 'High Serum IgG4 Concentrations in Patients with Sclerosing Pancreatitis'

Label	Title	KE	Year
leading article	High Serum IgG4 Concentrations in Patients with Sclerosing Pancreatitis	2564.4433	2001
A	Autoimmune related pancreatitis	1816.6444	2002
B	Close relationship between autoimmune pancreatitis and multifocal fibrosclerosis	69.1259	2003
C	Elevated IgG4 concentrations in serum of patients with Mikulicz's disease	70.3783	2004
D	Systemic extrapancreatic lesions associated with autoimmune pancreatitis.	467.8118	2005
E	Lymphoplasmacytic sclerosing pancreatitis and cholangitis	15.6051	2002
F	Acute tubulointerstitial nephritis associated with autoimmune-related pancreatitis	202.6705	2004
G	IgG4-related sclerosing cholangitis with and without hepatic inflammatory pseudotumor, and sclerosing pancreatitis-associated sclerosing cholangitis: do they belong to a spectrum of sclerosing pancreatitis?	34.1787	2004
H	Long-term prognosis of autoimmune pancreatitis with and without corticosteroid treatment	16.3812	2007
I	Autoimmune pancreatitis	35.0497	2005
J	Proposal for a new clinical entity, IgG4-positive multiorgan lymphoproliferative syndrome: analysis of 64 cases of IgG4-related disorders	85.6439	2009
K	Autoimmune pancreatitis	83.7350	2011
L	IgG4-related disease.	1956.8530	2012
M	Hydronephrosis associated with retroperitoneal fibrosis and sclerosing pancreatitis	12.7856	2002
N	Histopathological features of diagnostic and clinical relevance in autoimmune pancreatitis: a study on 53 resection specimens and 9 biopsy specimens	15.0222	2004
O	Consensus statement on the pathology of IgG4-related disease.	11.6600	2012

Table S4-20. Child articles details

The pioneering work of the idea tree is 'High Serum IgG4 Concentrations in Patients with Sclerosing Pancreatitis'. It was published in 2001, and it has already attracted 1,989 citations until 2021. A large number of high KE nodes are bred inside the idea tree, which makes the VD reach six.

S5 Measuring the development potential of publications in specific fields

Computer vision

NO.	Leading article	DPI
1	Rethinking Atrous Convolution for Semantic Image Segmentation	2.271
2	PointNet++: Deep Hierarchical Feature Learning on Point Sets in a Metric Space	2.095
3	The SYNTHIA Dataset: A Large Collection of Synthetic Images for Semantic Segmentation of Urban Scenes	2.067
4	ArcFace: Additive Angular Margin Loss for Deep Face Recognition	2.044
5	YOLO9000: Better, Faster, Stronger	2.018
6	You Only Look Once: Unified, Real-Time Object Detection	2.009
7	Deformable Convolutional Networks	1.995
8	Image Super-Resolution Using Deep Convolutional Networks	1.924
9	Dynamic Graph CNN for Learning on Point Clouds	1.906
10	Image-to-Image Translation with Conditional Adversarial Networkss	1.892

Table S5-1. Top ten publications in the field of computer vision appearing in the past ten years according to DPI

In the field of computer vision, the scientific publications in the first and third place of development potential is related to the semantic segmentation of images. With the urgent need for scene understanding in many practical applications such as current automatic driving and human-computer interaction, inferring corresponding semantic information from images has become a valuable application in the field of computer vision. In addition, there are also groundbreaking publication utilizing neural networks to process point cloud data (2), the creation (6) and improvement (5) of the current important YOLO model in object detection, and the seminal work utilizing deep learning to improve image resolution (8).

Natural language processing

NO.	Leading article	DPI
1	A Broad-Coverage Challenge Corpus for Sentence Understanding through Inference	2.183
2	Get To The Point: Summarization with Pointer-Generator Networks	1.976
3	Enriching Word Vectors with Subword Information	1.892
4	SQuAD: 100,000+ Questions for Machine Comprehension of Text	1.456
5	Listen, attend and spell: A neural network for large vocabulary conversational speech recognition	1.368
6	Google's Neural Machine Translation System: Bridging the Gap between Human and Machine Translation	1.319
7	Neural Machine Translation of Rare Words with Subword Units	1.223
8	Improving Neural Machine Translation Models with Monolingual Data	1.156
9	BERT: Pre-training of Deep Bidirectional Transformers for Language Understanding	1.150
10	XLNet: Generalized Autoregressive Pretraining for Language Understanding	1.091

Table S5-2. Top ten publications in the field of natural language processing appearing in the past ten years according to DPI

In the field of natural language processing, the improvement of technology is accompanied by the refresh of various rankings. In our list, the first work is the Multi-Genre Natural Language Inference (MNLI), and the fourth work is the Stanford Question Answering Dataset (SQuAD). This shows that the emergence of various types of datasets has a huge boost to the development of the field. In addition, the popular model Bert in recent years has proved that large models can improve the accuracy of natural language processing tasks and open up a new path for the development of the field, which makes relevant research appear in our list (9, 10).

Data mining

NO.	Leading article	DPI
1	Inductive Representation Learning on Large Graphs	1.598
2	Wide & Deep Learning for Recommender Systems	1.493
3	Membership Inference Attacks Against Machine Learning Models	1.307
4	Deep Neural Networks for YouTube Recommendations	1.260
5	Neural Collaborative Filtering	1.185
6	Deep Learning with Differential Privacy	1.185
7	Graph Attention Networks	1.163
8	Modeling Relational Data with Graph Convolutional Networks	1.073
9	XGBoost: A Scalable Tree Boosting System	1.047
10	Structural Deep Network Embedding	0.882

Table S5-3. Top ten publications in the field of data mining appearing in the past ten years according to DPI

In the field of data mining, the most promising publication is GraphSAGE, which is related to graph neural networks. GraphSAGE solves the problem that the graph convolutional network (GCN) is too slow when representing new nodes, and enables rapid deployment in the production environment to bring practical benefits. In the second place is a recommendation system framework based on deep learning proposed by Google (2). Google has applied this method to its Google Play app recommendation business, and it has also been imitated and applied by many companies. This shows its huge development potential.

References

1. Cormen TH, Leiserson CE, Rivest RL, Stein C. Introduction to algorithms. MIT press; 2022.

2. Symeonidis P, Tiakas E, Manolopoulos Y. Transitive node similarity for link prediction in social networks with positive and negative links. In: Proceedings of the fourth ACM conference on Recommender systems; 2010. p. 183–190.

3. Jeh G, Widom J. Simrank: a measure of structural-context similarity. In: Proceedings of the eighth ACM SIGKDD international conference on Knowledge discovery and data mining; 2002. p. 538–543.

4. Li A, Pan Y. Structural information and dynamical complexity of networks. IEEE Transactions on Information Theory. 2016;62(6):3290–3339.
